# High-Throughput Field Phenotyping of Leaves, Leaf Sheaths, Culms and Ears of Spring Barley Cultivars at Anthesis and Dough Ripeness

**DOI:** 10.3389/fpls.2017.01920

**Published:** 2017-11-07

**Authors:** Gero Barmeier, Urs Schmidhalter

**Affiliations:** Chair of Plant Nutrition, Department of Plant Sciences, Technical University of Munich, Freising, Germany

**Keywords:** deep phenotyping, morphological traits, passive sensor, phenomics, phenotyping, plant breeding, plant organs, proximal sensing

## Abstract

To optimize plant architecture (e.g., photosynthetic active leaf area, leaf-stem ratio), plant physiologists and plant breeders rely on destructively and tediously harvested biomass samples. A fast and non-destructive method for obtaining information about different plant organs could be vehicle-based spectral proximal sensing. In this 3-year study, the mobile phenotyping platform PhenoTrac 4 was used to compare the measurements from active and passive spectral proximal sensors of leaves, leaf sheaths, culms and ears of 34 spring barley cultivars at anthesis and dough ripeness. Published vegetation indices (VI), partial least square regression (PLSR) models and contour map analysis were compared to assess these traits. Contour maps are matrices consisting of coefficients of determination for all of the binary combinations of wavelengths and the biomass parameters. The PLSR models of leaves, leaf sheaths and culms showed strong correlations (*R*^2^ = 0.61–0.76). Published vegetation indices depicted similar coefficients of determination; however, their RMSEs were higher. No wavelength combination could be found by the contour map analysis to improve the results of the PLSR or published VIs. The best results were obtained for the dry weight and N uptake of leaves and culms. The PLSR models yielded satisfactory relationships for leaf sheaths at anthesis (*R*^2^ = 0.69), whereas only a low performance for all of sensors and methods was observed at dough ripeness. No relationships with ears were observed. Active and passive sensors performed comparably, with slight advantages observed for the passive spectrometer. The results indicate that tractor-based proximal sensing in combination with optimized spectral indices or PLSR models may represent a suitable tool for plant breeders to assess relevant morphological traits, allowing for a better understanding of plant architecture, which is closely linked to the physiological performance. Further validation of PLSR models is required in independent studies. Organ specific phenotyping represents a first step toward breeding by design.

## Introduction

Plant breeders, physiologists, and agronomists face a bottleneck in phenotyping (Winterhalter et al., 2011; White et al., 2012) due to a lack of efficient high-throughput field phenotyping methods. The result is a missing linkage between the genotype and phenotype (Furbank and Tester, 2011; Araus and Cairns, 2014). Current practices in field phenotyping, such as visual scoring or weighing biomass samples, are time consuming, labor intensive, costly and biased due to the experience of the researcher (Erdle et al., 2013; Kipp et al., 2014).

A fast and non-invasive method to obtain information about the characteristics of cultivars could be spectral proximal sensing (White et al., 2012; Erdle et al., 2013; Kipp et al., 2013). Vehicles (e.g., tractors, buggies) are particularly advantageous when a high number of genotypes and/or large plots of field trials need to be measured in the field. A further benefit of vehicles is the possibility of combining several sensors on a carrier vehicle to take measurements simultaneously (Winterhalter et al., 2011; Deery et al., 2014).

Studies have been performed for the spectral proximal sensing of cereal plant traits, such as the estimation of aerial biomass or the nitrogen status of spring and winter wheat (Erdle et al., 2013; Li et al., 2013a; Øvergaard et al., 2013; Xiu-liang et al., 2014; Bai et al., 2016), durum wheat (Ferrio et al., 2005), winter barley and rye, corn (Haboudane et al., 2004; Winterhalter et al., 2013) and spring barley (Yu et al., 2012; Bendig et al., 2014, 2015; Xu et al., 2014; Elsayed et al., 2015; Lausch et al., 2015; Tilly et al., 2015; Barmeier and Schmidhalter, 2016; Rischbeck et al., 2016). Still data analysis remains a major challenge. While many authors rely on various vegetation indices (VI) (such as the: NDVI, REIP, PRI, WI, SAVI, TCARI) (Behrens et al., 2006; Yu et al., 2012; Erdle et al., 2013; Li et al., 2013a; Bendig et al., 2015; Elsayed et al., 2015; Tilly et al., 2015), additional methods, such as “contour map analysis” and “partial least squares regression” (PLSR), have been highlighted as particularly interesting to optimize data analysis. These methods were used by Hansen and Schjoerring (2003) to detect the biomass and nitrogen status, by Li et al. (2013a) to estimate the nitrogen content, and by Elsayed et al. (2015) and Rischbeck et al. (2016) to predict drought stress and grain yield in barley.

Previously, most authors have investigated biomass parameters such as fresh and dry weight or aboveground nitrogen uptake that was subjected to varying management actions or reflecting combined growth stages. By contrast, in plant breeding nurseries, uniform management is conducted, thus lowering the variance due to agronomic treatments. Hence, breeders require spectral sensors and algorithms that allow to detect more subtle differences among cultivars independent of agronomic management practices or dependent on specific growth stages.

Acquaah (2012) has reported on the various points of view of plant breeders regarding the importance of different plant organs. In addition to grains, there are other important plant organs, such as culms for the production of straw. Furthermore, culms are the most important storage organs of assimilates for translocation processes after anthesis (Bidinger et al., 1977; Mirosavljevic et al., 2015). Unadapted or stressed cultivars particularly rely on the dry matter and nitrogen reserves of culms (Przulj and Momcilovic, 2001a,b). Knowledge regarding the characteristics of the leaves of a cultivar or variety is important for plant breeders when optimization of the photosynthetic active area is intended (Haboudane et al., 2004). Zhu et al. (2010) mentioned that an improvement of the leaf area and architecture may avoid saturation effects of individual leaves and support higher grain yields. Additionally, leaves act as a sink for nutrients as well as a source of proteins and are therefore important for grain yield formation (Acquaah, 2012). The role of leaf sheaths as a vertical part of leaves has not been widely reported in the literature. Schnyder (1993) characterized leaf sheaths as long-term storage for carbohydrates that are influenced by environmental conditions. In this study were cultivars evaluated that accumulated up to 20 kg N ha^−1^ in leaf sheaths at anthesis (Supplementary Tables 1, 2).

The question is, how precisely can these plant organs be detected by spectral sensors?

Active and passive spectrometers were evaluated in this study. While passive spectrometers depend on sunlight as its source of light, active sensors use independent light sources, such as LED or Xenon lamps (Erdle et al., 2011). The advantage of active sensors is that they can be applied during changing light conditions or at night without any effect on their readings (Hatfield et al., 2008; Kim et al., 2012; Kipp et al., 2014). However, the bidirectional passive spectrometer used in this study is equipped with two detectors, one measures global radiation as a reference signal, and the second one measures the reflectance of the plant canopy to avoid effects due to changing light conditions (Mistele and Schmidhalter, 2010).

Technical comparisons among different sensor systems for the prediction of specific plant traits have been performed multiple times. Erdle et al. (2011), Winterhalter et al. (2013), Elsayed et al. (2015) and Becker and Schmidhalter (2017) evaluated active and passive sensors in winter wheat, corn, and spring barley, respectively. The performance of active sensors under changing environmental conditions was evaluated by Kim et al. (2012) for the GreenSeeker and Kipp et al. (2014) for the GreenSeeker, CropCircle, and AFS N-Sensor.

The potential of spectral proximal sensors to detect, in addition to the total aerial biomass and nitrogen content, the characteristics of different plant organs was first shown by Erdle et al. (2013). In contrast to Erdle et al., who considered later growth stages in winter wheat, this study focuses on spring barley during anthesis and dough ripeness. These stages revealed to be particularly interesting to predict yield and yield parameters in our previous work. A set of 30–34 spring barley cultivars was separated into leaves, leaf sheaths, culms and ears at anthesis and dough ripeness. Sensor measurements were made by using two commercially available and two custom built spectral sensors. The aims of this study were to perform (i) a comparison of different spectral proximal sensors and (ii) a comparison of published vegetation indices, contour maps and PLSR to assess leaves, leaf sheaths, culms and ears in spring barley.

## Materials and methods

### Field experiments

Field experiments were conducted at the Dürnast research station of the Technical University of Munich in Germany (11°41′60″E, 48°23′60″N) from 2013 to 2015. The soil is characterized as a mostly homogeneous cambisol of silty clay loam. The annual precipitation is ~800 mm, and the average temperature is 7.8°C. This 3-year study encompassed 30–34 spring barley (*Hordeum vulgare* L.) in a randomized block design with four replicates (Table 1). The plots consisted of 12 rows, 10.9 m in length. The fungicide and fertilization treatments followed local recommendations.

**Table 1:** Overview of spring barley cultivars grown in different years.

**Cultivar**	**Usage**	**2013**	**2014**	**2015**
Aspen	Malting	X	X	X
Barke	Malting	X	X	X
Baronesse	Malting	X	X	X
Br8993a3	–	X		
Braemar	Malting	X	X	X
Calcule	Fodder	X	X	X
Carina	Malting	X	X	X
Djamila	Fodder	X	X	X
Eunova	Fodder	X	X	X
Grace	Malting	X	X	X
Hora^*^	Human food			X
IPZ 24727	Malting	X	X	X
Irina	Malting	X	X	X
Lawina^*^	Human food	X		
Mackay [AUS]	Malting	X	X	X
Marthe	Malting	X	X	X
Melius	Malting	X	X	X
Paradiesgerste^*^	Human food			X
Pirona^*^	Human food		X	X
Power	Malting	X	X	X
Quench	Malting	X	X	X
Salome	Malting	X	X	X
Scarlett	Malting	X	X	X
Shakira	Malting	X	X	X
Sissy	Malting	X	X	X
Solist	Malting	X	X	X
Streif	Fodder	X	X	X
Trumpf/Triumph	Malting	X	X	X
Union	Malting	X	X	X
Ursa	Malting	X	X	X
UTA	Malting			X
Vespa	Fodder	X	X	X
Volla	Malting	X	X	X
Wiebke	Malting	X	X	X

* *Hull-less barley*.

### Biomass sampling

Biomass sampling was performed at anthesis (ZS 65) and at soft dough ripeness (ZS85) (Zadoks et al., 1974) by harvesting 30 plants from each plot randomly. The plants were separated into ears, leaves, leaf sheaths (in 2014 and 2015) and culms. The biomass samples were oven dried at 60°C for 2 days to achieve a constant moisture content and then weighed. The N content was detected by mass spectrometry using an Isotope Radio Mass Spectrometer with an ANCA SL 20–20 preparation unit (Europe Scientific, Crewe, UK), and N uptake was calculated by multiplying the plant dry weight by the total N content.

### Spectral measurements

**Figure 1:**
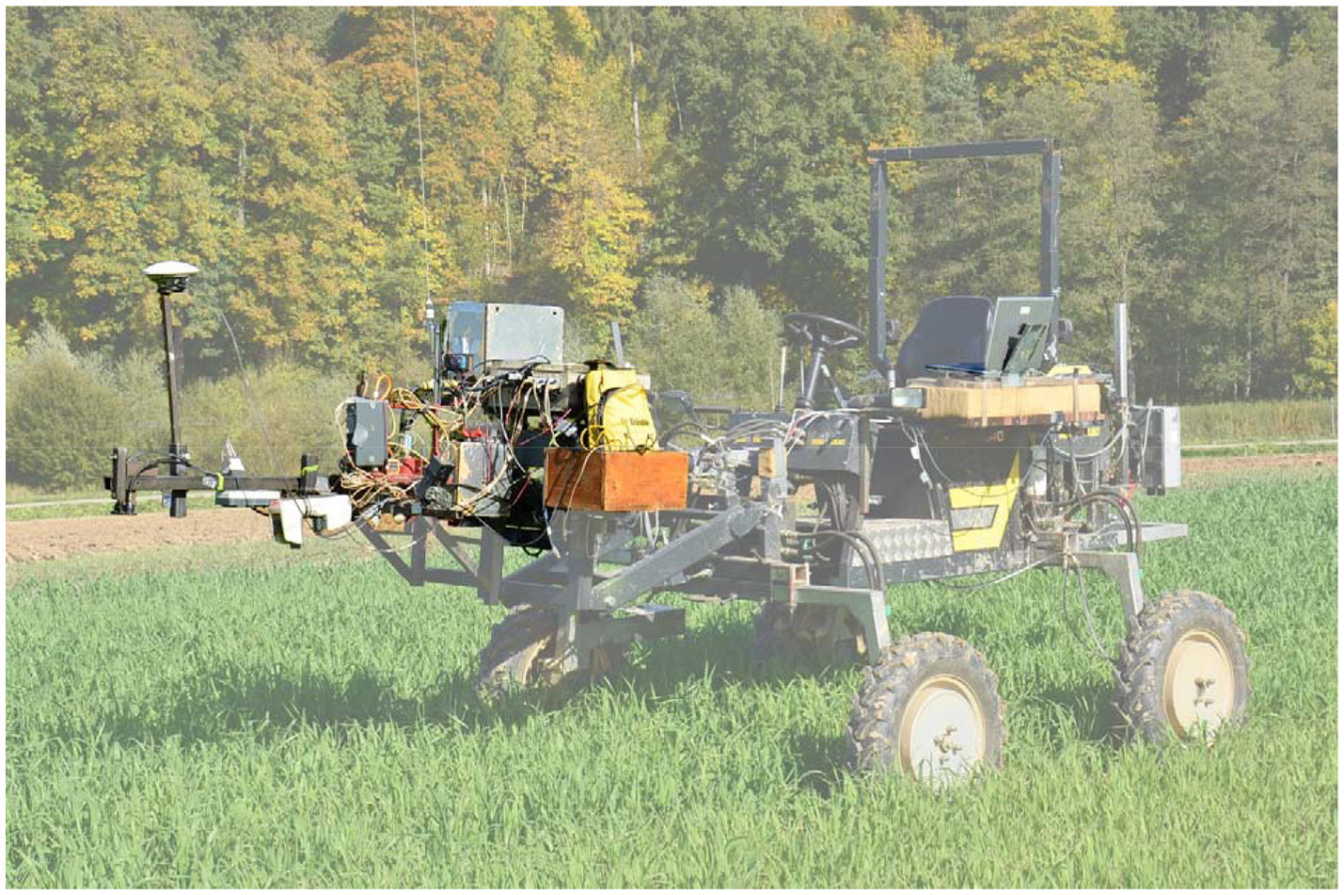
Phenotyping platform PhenoTrac 4 of the Chair of Plant Nutrition from the Technical University of Munich.

The sensor system consisted of three active spectral sensors and a passive hyperspectral sensor that were mounted aligned in a row on a frame on the mobile phenotyping vehicle PhenoTrac 4 from the Chair of Plant Nutrition at the Technical University of Munich (Figure 1). This phenotyping platform is a small and lightweight diesel-powered tractor (850 kg) with a ground clearance of 1 m and a speed of 6 km h^−1^. The sensor carrier was positioned 1 m above the plant canopy, and measurements were taken under clear sky conditions at noon. While collecting information in the field, the sensor outputs were co-recorded along with GPS coordinates from the TRIMBLE RTK-GPS (Trimble, Sunnyvale, CA, USA). The passive hyperspectral bidirectional reflectance sensor contains two Zeiss MMS1 silicon diode array spectrometers with a spectral detection range from 300 to 1,700 nm and has a bandwidth of 3.3 nm (Mistele and Schmidhalter, 2008), but was restricted in this study to 1,000 nm. One spectrometer was linked to a diffuser that detected solar radiation as a reference signal. The second spectrometer measured the canopy reflectance with a field of view (FOV) of 12° that was circular in shape, resulting in a scanned area of 0.28 m^2^ and covering an area of 5.45 m^2^ along the plot. The passive spectrometer was calibrated before each measurement using a gray standard. The active spectral sensor GreenSeeker RT100 (NTech Industries, Ukiah, CA, USA) uses two LEDs as a light source and detects the reflection of both in the VIS (656 nm, ~25 nm band width) and NIR (774 nm, ~25 nm band width) spectral regions. As a second active spectral sensor, an active flash sensor (AFS) was used that was similar to the N-Sensor ALS® (YARA International, ASA) with a flashing xenon light as a light source, producing a spectral range of 650–1,100 nm with 10 flashes per second. In this experiment, filters similar to those of the YARA ALS® system were chosen: 730, 760, 900, and 970 nm (Erdle et al., 2011). The third active spectral sensor was a CropCircle ACS-470® (Holland Scientific, Inc., Lincoln, NE), which emits white light (light source: ~400 to 800 nm), with a selection of filters for wavelengths of 670, 730, and 760 nm. The CropCircle was only used in 2013 and 2015. With reference to the manufacturers' information, the active sensors were calibrated before delivery and no additional calibration was required.

Table 2 shows the vegetation indices selected for this experiment.

**Table 2:** Selected vegetation indices of the four sensor systems used.

**Device**	**Vegetation index**	**References**
GreenSeeker	R774/R656	
	NDVI	Rouse et al., 1974
CropCircle	R730/R670	
	R760/R730	Mistele and Schmidhalter, 2010
	R760/R670	Mistele and Schmidhalter, 2008
	NDVI	Rouse et al., 1974
AFS	R760/R730	Mistele and Schmidhalter, 2010
	R900/R970	Peñuelas et al., 1993
Passive spectrometer	R780/R550	Mistele and Schmidhalter, 2008
	R780/R670	Pearson et al., 1972
	R780/R700	Guyot et al., 1988
	R760/R670	Erdle et al., 2011
	R760/R730	Mistele and Schmidhalter, 2010
	R780/R740	Mistele and Schmidhalter, 2010
	R900/R970	Peñuelas et al., 1993
	REIP	Guyot et al., 1988
	NDVI	Rouse et al., 1974

### Statistical analysis

The main effects and interactions between cultivars and years were tested using a two-way analysis of variance (ANOVA) by using the model: y_ija_ = y_i_ + (yr)_ia_ + g_j_ + (gy)_jj_ + ε_ija_.

Where y_ija_ is the observation in the year i, of the genotypes j in the replicate a, and yi the effect of the year i, and (yr)_ia_ the interaction between the year i and the replicate a, and g_j_ the effect of genotype j, and the interaction between the i-th year and the j-th genotype. We consider all factors as fixed. In the ANOVA, the effect of the year was tested against the year^*^replication effect, while the genotype and the genotype^*^year interaction was tested against the overall residual (McIntosh, 1983).

Linear regression between the data obtained from the sensors and destructive measurements were calculated by using R version 3.1.2. (R Core Team, 2016).

In order to find new wavelength combinations for an optimized vegetation index the R package “lattice” (http://lattice.r-forge.r-project.org/) (R version 3.0.2, R Core Team, 2016) was used to calculate contour maps. Contour maps are matrices consisting of coefficients of determination for all binary combinations of wavelengths and the biomass parameters.

PLSR was calculated to find improved relationships between canopy reflectance and the biomass parameters by using The Unscrambler® X 10.3 (Camo Software AS, Oslo, Norway). PLSR is a multivariate statistical method used to find “latent” structures in the wavelength spectra (X) that best predict the measured parameter (Y). This method is advantageous when dependent (response) variables need to be predicted from large datasets of predictor variables. The dataset is reduced to a few “principal components” (PC) or “factors” that are used for prospective predictions of the response variables. A detailed description of PLSR can be found in Esbensen et al. (2002).

In this study, PLSR was used to model the correlation with individual replicate means (*n* = 34 × 4) as well as well as with genotypic means (*n* = 34) between the full canopy reflectance spectrum (predictor variables) from the passive spectrometer in a waveband region between 400 and 1000 nm and the biomass parameters (response variables). We highlight in this work particularly the performance of non-destructive sensing to detect the plotwise differentiation of specific plant organs. Averaging genotypic differences will be particularly useful with an increased panel of genotypes being required for an enhanced PLS analysis, but is presented as well.

All of the spectral data used to calculate the PLSR models were corrected for light scattering using Standard Normal Variate Transformation (SNV). The dataset was randomly separated into subsets, with 2/3 of the observations for calibration and 1/3 for validation of the models combining the three-year data. The optimum number of PC selected in the PLS analyses were based on the first clear V-minimum or a break from monotonically decreasing variance, i.e., where the prediction error is minimized.

To assess the quality of the PLSR models, the vegetation indices and the optimized vegetation indices obtained from the contour maps, root mean square errors (RMSE) and the Pearson coefficients of determination (*R*^2^) were compared. Finding models with a combination of a low RMSE and a high *R*^2^ was the target objective.

To further test the predictive performance of the PLSR models an independent data set of 13 wheat cultivars was used which were grown at 160 and 220 kg N ha^−1^ in 2015 and at 100, 160, and 220 kg N ha^−1^ in 2016, encompassing the same organ assessments. This data set was chosen, since no independent data set providing this information from barley cultivars was available. The barley PLSR models were therefore used to predict plant biomass, leaf, and culm biomass as well as the organ-specific nitrogen uptake of wheat cultivars at anthesis. The model performance was tested for individual years, since their information differed statistically. This allows for the classification of the best performing cultivars, independent of the year, since only a relative comparison is strived for.

## Results

### Agronomic parameters and weather conditions

The year 2014 was the most favorable for spring barley due to an average temperature of ~18.3°C during anthesis and evenly distributed precipitation. By contrast, unfavorable weather conditions between germination and anthesis led to a dry weight that was reduced by ~42% in 2013. The number of ears per square meter was comparable in all 3 years, with ~635 ears sqm^−1^. With regard to the total dry weight and total N uptake, significantly lower values were observed in cultivars processed for human nutrition in all years. An exception to this result was the cultivar Pirona, which accumulated the highest total dry weight of all cultivars while having the lowest ear dry weight in 2014.

### Correlations between traits

Correlations between plant organs and the final grain yield are given in Table 3. The highest coefficients of determination were found for the number of “ears sqm^−1^” in all 3 years. While the individual plant organs showed low or medium correlation to grain yield, the total plant dry weight indicated consistently higher coefficients of determination up to *R*^2^ = 0.62. The lowest values were obtained for leaf sheaths.

**Table 3:** Coefficients of determination (*R*^2^) between plant organs and grain yield (*p* ≤ 0.01) with individual replicate means (n 34 × 4).

**Year**		**2013**	**2014**	**2015**
	Ears/m^2^	0.65^**^	0.62^**^	0.70^**^
Anthesis	DW leaves kg/ha	0.22^**^	0.21^**^	0.51^**^
	DW leaf sheaths kg/ha		0.09	0.27^**^
	DW culms kg/ha	0.34^**^	0.43^**^	0.55^**^
	DW total	0.34^**^	0.42^**^	0.58^**^
	DW leaves + leaf sheaths kg/ha		0.3^**^	0.52^**^
	DW leaves + culms	0.34^**^	0.40^**^	0.57^**^
	DW leaf sheaths + culms kg/ha		0.45^**^	0.56^**^
	N uptake leaves kg/ha	0.17	0.32^**^	0.54^**^
	N uptake leaf sheaths kg/ha		0.13^**^	0.29^**^
	N uptake culms kg/ha	0.31^**^	0.45^**^	0.55^**^
	N uptake total kg/ha	0.33^**^	0.45^**^	0.61^**^
	N uptake leaves + leaf sheaths kg/ha		0.38^**^	0.58^**^
	N uptake leaves + culms kg/ha	0.33^**^	0.43^**^	0.61^**^
	N uptake leaf sheaths + culms kg/ha		0.46^**^	0.56^**^
Dough ripeness	DW ears kg/ha	0.54^**^	0.42^**^	0.56^**^
	DW leaves kg/ha	0.31^**^	0.25^**^	0.54^**^
	DW leaf sheaths kg/ha		0.21^**^	0.15^**^
	DW culms kg/ha	0.25^**^	0.19^**^	0.4^**^
	DW total	0.51^**^	0.43^**^	0.62^**^
	DW ears + leaves kg/ha	0.54^**^	0.47^**^	0.61^**^
	DW ears + leaf sheaths kg/ha		0.43^**^	0.56^**^
	DW ears + culms kg/ha	0.51^**^	0.42^**^	0.6^**^
	DW ears + leaves + leaf sheaths kg/ha		0.47^**^	0.6^**^
	DW ears + leaves + culms kg/ha		0.42^**^	0.62^**^
	DW ears + leaf sheaths + culms kg/ha		0.43^**^	0.61^**^
	DW leaves + leaf sheaths kg/ha		0.33^**^	0.41^**^
	DW leaves + culms kg/ha	0.28^**^	0.21^**^	0.44^**^
	DW leaves + leaf sheaths + culms kg/ha		0.24^**^	0.49^**^
	DW leaf sheaths + culms kg/ha		0.22^**^	0.46^**^
	N uptake ears kg/ha	0.45^**^	0.44^**^	0.36^**^
	N uptake leaves kg/ha	0.16	0.27^**^	0.49^**^
	N uptake leaf sheaths kg/ha		0.29^**^	0.28^**^
	N uptake culms kg/ha	0.24^**^	0.07	0.25^**^
	N uptake total kg/ha	0.49^**^	0.39^**^	0.52^**^
	N uptake ears + leaves kg/ha	0.45^**^	0.48^**^	0.46^**^
	N uptake ears + leaf sheaths kg/ha		0.46^**^	0.39^**^
	N uptake ears + culms kg/ha	0.50^**^	0.07^**^	0.41^**^
	N uptake ears + leaves + leaf sheaths kg/ha		0.49^**^	0.48^**^
	N uptake ears + leaves + culms kg/ha		0.38^**^	0.49^**^
	N uptake ears + leaf sheaths + culms kg/ha		0.38^**^	0.44^**^
	N uptake leaves + leaf sheaths kg/ha		0.32^**^	0.54^**^
	N uptake leaves + culms kg/ha	0.27^**^	0.15	0.47^**^
	N uptake leaves + leaf sheaths + culms kg/ha		0.18	0.56^**^
	N uptake leaf sheaths + culms kg/ha		0.12	0.47^**^

*Units of organs are expressed as kg ha^−1^*.

** *Denotes significance at the 0.01 level*.

A weak significant relationship (*R*^2^ = 0.27) was observed between leaf dry weight and culm dry weight at anthesis, increasing to *R*^2^ = 0.75 at dough ripeness. No relationships were observed between leaf dry weight and dry weights of leaf sheaths and ears either at both growth stages or at dough ripeness, respectively. Culm dry weight was not related to ear dry weight and only weakly related to leaf sheath dry weight.

No relationship was observed between leaf N uptake and the N uptake of leaf sheaths and ears, whereas leaf N uptake was related to the culm N uptake with *R*^2^ = 0.42 and 0.67 at anthesis and dough ripeness, respectively. The N uptake of ears was related to the N uptake of leaf sheaths and culms with *R*^2^ = 0.38 and 0.51, respectively, at dough ripeness.

### Anova of the organ specific dry weights and N uptake, and sensor measurements at anthesis and dough ripeness

Cultivars differed significantly in the organ-specific dry weights and N-uptake of leaves, culms, and leaf sheaths as well as the total biomass dry weight at anthesis (Table 4).

**Table 4:** *F*-values of the ANOVA of the organ specific dry weight and N uptake, and sensor measurements at anthesis (*p* ≤ 0.01).

***F*-values**	**Traits**	**Cultivars**	**Year**	**CxY**
df		33	2	55
	DWleaves	4.08^**^	16.3^**^	1.87^**^
	DWculms	2.6^**^	5.08^**^	1.47^*^
	DWtotal	2.41^**^	8.99^**^	1.34
	NupLeaves	2.53^**^	30.99^**^	1.58^*^
	NupCulms	1.94^**^	25.45^**^	1.30
	NupTotal	1.71^*^	2.23^**^	1.27
	GS774_656	2.35^**^	143.46^**^	1.49^*^
	GSNDVI	2.23^**^	165.3^**^	1.01
	ALS760_730	2.67^**^	195.25^**^	1.22
	ALS900_970	3.05^**^	150.72^**^	1.7^**^
	PS780_550	2.11^**^	41.04^**^	0.97
	PS780_670	1.59^*^	174.32^**^	0.88
	PS780_700	1.98^**^	78.46^**^	0.87
	PS760_730	2.15^**^	35.36^**^	0.96
	PS780_740	2.11^**^	3.5^**^	1.15
	PS900_970	2.37^**^	20.64^**^	1.38^*^
	PSREIP	2.47^**^	15.67^**^	1.24
	PSNDVI	2.65^**^	117.58^**^	1.22
df		33	1	27
	CC730_670	1.63^*^	2.38^**^	0.91
	CC760_730	2.28^**^	16.93^**^	0.84
	CC760_670	1.58^*^	37.53^**^	0.82
	CCNDVI	2.2^**^	7.78^**^	0.8
df		31	1	28
	DWsheaths	2.26^**^	16.64^**^	1.17
	NupSheaths	2.12^**^	42.42^**^	1.3

* p ≤ 0.05 and

** *p ≤ 0.01*.

Significant differences among cultivars were also observed at dough ripeness for the dry weights of ears, leaves and culms, except for the total plant dry weight. N uptake of leaves and culms differed as well, whereas ear N uptake and total N uptake did not differ at dough ripeness (Table 5).

**Table 5:** *F*-values of the ANOVA of the organ specific dry weight and N uptake, and sensor measurements at dough ripeness (*p* ≤ 0.01).

***F*-values**	**Traits**	**Cultivars**	**Year**	**CxY**
df		34	2	54
	DWears	2.66^**^	11.44^**^	1.58^*^
	DWleaves	2.67^**^	6.12^**^	1.9^**^
	DWculms	2.97^**^	24.65^**^	1.9^**^
	DWtotal	1.37	1.7^**^	1.45^*^
	NupEars	1.25	9.35^**^	1.19
	NupLeaves	2.27^**^	10.31^**^	2.08^**^
	NupCulms	3.6^**^	28.7^**^	2.18^**^
	NupTotal	1.07	0.52^**^	1.22
	GS774_656	1.55^*^	36.42^**^	2.12^**^
	GSNDVI	1.43	70.86^**^	1.79^**^
	ALS760_730	2.3^**^	64.42^**^	1.64^**^
	ALS900_970	1.75^**^	289.79^**^	2.05^**^
	PS780_550	2.32^**^	67.09^**^	1.38^*^
	PS780_670	1.19	54.59^**^	1.25
	PS780_700	1.52^*^	69.55^**^	1.23
	PS760_730	1.63^*^	53.23^**^	1.31
	PS780_740	2.04^**^	7.62^**^	1.77^**^
	PS900_970	2.17^**^	91.67^**^	1.35
	PSREIP	2.56^**^	70.38^**^	1.95^**^
	PSNDVI	1.67^*^	120.17^**^	1.2
df		34	1	26
	CC730_670	2.05^**^	16.46^**^	1.89^**^
	CC760_730	2.1^**^	46.25^**^	0.87
	CC760_670	1.64^*^	67.45^**^	1.39
	CCNDVI	1.86^**^	41.97^**^	1.11
df		31	1	28
	DWsheaths	1.17	2.48^**^	0.84
	NupSheaths	1.4	3.52^**^	1.11

* *p ≤ 0.05 and *.

** *p ≤ 0.01*.

All sensors differentiated cultivars at anthesis (Table 4). Spectral differentiation at dough ripeness was enabled by the ALS sensor, the CropCircle and five of the tested indices from the hyperspectral sensor (Table 5), whereas no differentiation was obtained for the GreenSeeker.

### Detection of the dry weight and N uptake of leaves

An overview of the descriptive statistics of the dry weight and N uptake of leaves is given in the Supplementary Table 1. Low dry weight and N uptake were observed due to unfavorable weather conditions in 2013. The highest leaf biomass values were observed for Pirona and the lowest for Hora. Both cultivars have hull-less grains and are processed for human nutrition; however, Pirona was the tallest cultivar, whereas Hora was one of the smallest. A comparable tendency was observed for culms.

Table 6 shows the results of the plotwise PLSR, and Table 7 shows the linear regressions of the vegetation indices obtained for each sensor. Additional information depicting the genotype-wise differentiation by PLSR is shown in Supplementary Tables 5, 6.

**Table 6:** Results of the plotwise PLSR analysis of the dry weight and N uptake of leaves used for calibration and validation, the genotype-wise differentiation is contained in the Supplementary Tables 5, 6.

		**PC**	**Calibration**				**Validation**			
			**Slope**	**Offset**	**RMSE**	***R*^2^**	**Slope**	**Offset**	**RMSE**	***R*^2^**
Anthesis	DW leaves (kg/ha)	4	0.62	383.9	160.36	0.62	0.60	373.6	181.29	0.65
	N uptake leaves (kg/ha)	4	0.74	5.61	5.20	0.74	0.76	4.71	5.23	0.76
Dough ripeness	DW leaves (kg/ha)	4	0.56	384	168.04	0.56	0.52	426	181.97	0.52
	N uptake leaves (kg/ha)	5	0.66	3.8	2.85	0.66	0.58	4.4	3.52	0.62

**Table 7:** Results of linear regression of the dry weight and N uptake of leaves showing the tested vegetation indices from three active and one passive spectral sensor at anthesis and dough ripeness.

**Leaves (kg/ha)**			**CropCircle**				**GreenSeeker**		**AFS**	
			**R730/R670**	**R760/R730**	**R760/R670**	**NDVI**	**R774/R656**	**NDVI**	**R760/R730**	**R900/R970**
Dry weight	Anthesis	Slope	0.0007	0.0005	0.0024	0.0003	0.0023	0.0003	0.0003	0.0000
		Offset	0.81	1.22	−0.26	0.11	0.93	0.22	1.20	0.97
		RMSE	1,125.63	1,125.24	1,126.65	1,126.30	1,049.48	1,050.16	1,049.23	1,049.45
		*R*^2^	0.39^**^	0.26^**^	0.26^**^	0.36^**^	0.49^**^	0.44^**^	0.44^**^	0.14^**^
	Dough ripeness	Slope	0.0008	0.0003	0.0021	0.0003	0.0015	0.0002	0.0003	0.0000
		Offset	0.74	1.26	−0.01	0.08	1.40	0.22	1.21	1.00
		RMSE	907.84	907.34	908.55	908.46	914.19	915.33	914.37	914.58
		*R*^2^	0.19^**^	0.08	0.10	0.13^*^	0.19^**^	0.16^*^	0.31^**^	0.01
N uptake	Anthesis	Slope	0.0175	0.0188	0.1002	0.0095	0.0762	0.0090	0.0097	0.0026
		Offset	1.07	1.32	0.07	0.21	1.56	0.28	1.32	0.96
		RMSE	27.22	26.99	28.06	28.05	22.78	24.17	23.23	23.56
		*R*^2^	0.47^**^	0.66^**^	0.76^**^	0.6^**^	0.63^**^	0.63^**^	0.43^**^	0.50^**^
	Dough ripeness	Slope	0.0563	0.0362	0.2065	0.0266	0.1027	0.0162	0.0145	0.0034
		Offset	0.78	1.18	−0.39	0.06	1.48	0.22	1.27	0.98
		RMSE	27.22	26.99	28.06	28.05	22.78	24.17	23.23	23.56
		*R*^2^	0.59^**^	0.52^**^	0.54^**^	0.56^**^	0.45^**^	0.43^**^	0.48^**^	0.14^*^
**Leaves (kg/ha)**			**Passive spectrometer**							
			**R780/R550**	**R780/R670**	**R780/R700**	**R760/R730**	**R780/R740**	**R900/R970**	**REIP**	**NDVI**
Dry weight	Anthesis	Slope	0.0028	0.0186	0.0029	0.0002	0.0001	0.0001	0.0037	0.0002
		Offset	2.51	−5.75	1.04	1.13	1.13	1.12	715.69	0.57
		RMSE	1,047.97	1,055.61	1,049.37	1,049.30	1,049.30	1,049.31	420.36	1,049.83
		*R*^2^	0.52^**^	0.56^**^	0.55^**^	0.54^**^	0.28^**^	0.47^**^	0.4^**^	0.49^**^
	Dough ripeness	Slope	0.0025	0.0113	0.0023	0.0002	0.0001	0.0002	0.0045	0.0003
		Offset	2.30	−1.68	1.09	1.10	1.12	1.06	713.79	0.42
		RMSE	913.32	917.05	914.48	914.49	914.46	914.52	305.32	915.13
		*R*^2^	0.33^**^	0.4^**^	0.33^**^	0.32^**^	0.25^**^	0.37^**^	0.17^*^	0.29^**^
N uptake	Anthesis	Slope	0.0844	0.5583	0.0927	0.0077	0.0013	0.0027	0.1131	0.0076
		Offset	3.49	0.65	1.88	1.20	1.16	1.15	716.90	0.64
		RMSE	20.90	16.15	22.38	23.34	23.38	23.39	776.29	23.85
		*R*^2^	0.53^**^	0.59^**^	0.67^**^	0.63^**^	0.16^*^	0.46^**^	0.44^**^	0.6^**^
	Dough ripeness	Slope	0.1467	0.6337	0.1390	0.0151	0.0036	0.0093	0.3164	0.0191
		Offset	2.76	0.88	1.50	1.13	1.13	1.10	714.06	0.48
		RMSE	20.90	16.15	22.38	23.34	23.38	23.39	776.29	23.85
		*R*^2^	0.54^**^	0.6^**^	0.58^**^	0.58^**^	0.4^**^	0.51^**^	0.4^**^	0.51^**^

Significant R^2^-values are indicated at

* p ≤ 0.05 and

** *p ≤ 0.01*.

Fair relationships were found for the leaf dry weight, whereas for the leaf N uptake, slightly better results were found. Compared to the PLSR, the vegetation indices showed much higher RMSEs together with lower coefficients of determination. For the detection of the dry weight and N uptake of leaves, the R780/R670 vegetation index was found to be most promising, no further improvement was obtained from the Contour Map analysis evaluating all binary combinations of wavelengths (data not shown). Slight advantages for the passive spectrometer for detecting leaf dry weight at anthesis were observed; however, the CropCircle performed comparably well for measuring leaf N uptake.

### Detection of the dry weight and N uptake of leaf sheaths

The descriptive statistics of the leaf sheaths can be found in the Supplementary Table 2. In 2015, an ~35% higher dry weight and 66% higher N uptake at anthesis was found compared to that in 2014. The cultivars Shakira and Pirona showed the lowest dry weight and N uptake in both years, and IPZ 24727 showed the highest values.

The results of the plotwise PLSR are presented in Table 8 and the results for the genotype-wise differentiation by PLSR are contained in Supplementary Tables 5, 6. Good relationships were found for the N uptake of leaf sheaths; however, the dry weight of this plant organ was barely detectable by sensing at dough ripeness.

**Table 8:** Results of the plotwise PLSR analysis of the dry weight and N uptake of leaf sheaths used for calibration and validation, the genotype-wise differentiation is contained in the Tables 5, 6.

		**PC**	**Cal**				**Val**			
			**Slope**	**Offset**	**RMSE**	***R*^2^**	**Slope**	**Offset**	**RMSE**	***R*^2^**
Anthesis	DW leaf sheaths (kg/ha)	2	0.46	298.6	144.59	0.46	0.40	321.4	166.38	0.42
	N uptake leaf sheaths (kg/ha)	4	0.76	1.97	2.32	0.76	0.79	1.30	2.64	0.69
Dough ripeness	DW leaf sheaths (kg/ha)	3	0.25	562	182.71	0.25	0.20	617	218.22	0.21
	N uptake leaf sheaths (kg/ha)	5	0.49	4.3	2.42	0.49	0.54	4.0	2.16	0.50

Linear regressions between the leaf sheaths and vegetation indices are shown in Table 9. For the vegetation indices, only weak relationships with high RMSEs were observed. The highest coefficient of determination (*R*^2^ = 0.38) was delivered by the AFS sensor for N uptake at anthesis, with no further improvement delivered by the the Contour Map analysis (data not shown).

**Table 9:** Results of linear regression analysis of the dry weight and N uptake of leaf sheaths, showing the tested vegetation indices from three active and one passive spectral sensor at anthesis and dough ripeness.

**Leaf sheaths (kg/ha)**			**CropCircle**				**GreenSeeker**		**AFS**	
			**R730/R670**	**R760/R730**	**R760/R670**	**NDVI**	**R774/R656**	**NDVI**	**R760/R730**	**R900/R970**
Dry weight	Anthesis	Slope	0.0006	0.0005	0.0018	0.0003	−0.0013	−0.0001	−0.0003	0.0000
		Offset	1.04	1.48	1.41	0.25	4.45	0.61	1.75	1.02
		RMSE	576.57	576.16	576.22	577.29	424.44	427.04	426.27	426.76
		*R*^2^	0.17^*^	0.25^**^	0.20^**^	0.22^**^	0.11	0.11	0.16^*^	0.23^**^
	Dough ripeness	Slope	0.0382	0.0108	0.0812	0.0125	0.0941	0.0121	−0.0033	0.0071
		Offset	1.28	1.64	2.12	0.35	2.48	0.41	1.50	0.99
		RMSE	547.36	547.06	546.68	548.10	474.97	476.42	475.65	476.01
		*R*^2^	0.16^*^	0.07	0.14^*^	0.16^*^	0.14^*^	0.16^*^	0.00	0.24^**^
N uptake	Anthesis	Slope	0.0345	0.0245	0.1003	0.0144	−0.0879	−0.0087	−0.0201	0.0017
		Offset	1.06	1.50	1.49	0.27	4.44	0.61	1.75	1.03
		RMSE	7.33	6.99	6.88	8.00	4.84	5.73	5.20	5.51
		*R*^2^	0.16^*^	0.23^**^	0.18^*^	0.20^**^	0.24^**^	0.27^**^	0.37^**^	0.29^**^
	Dough ripeness	Slope	0.0004	0.0001	0.0008	0.0001	0.0009	0.0001	0.0000	0.0001
		Offset	1.20	1.62	1.95	0.33	2.34	0.39	1.53	0.98
		RMSE	5.00	4.71	4.48	5.66	3.76	4.86	4.22	4.50
		*R*^2^	0.23^**^	0.12	0.20^**^	0.25^**^	0.25^**^	0.28^**^	0.01	0.41^**^
**Leaf sheaths (kg/ha)**			**Passive spectrometer**							
			**R780/R550**	**R780/R670**	**R780/R700**	**R760/R730**	**R780/R740**	**R900/R970**	**REIP**	**NDVI**
Dry weight	Anthesis	Slope	−0.0010	−0.0132	−0.0010	0.0000	0.0000	0.0000	0.0002	−0.0001
		Offset	6.31	23.55	5.03	1.44	1.19	1.25	719.72	0.91
		RMSE	423.19	411.94	424.05	426.48	426.65	426.61	531.00	426.84
		*R*^2^	0.03	0.15	0.05	0.01	0.00	0.05	0.00	0.08
	Dough ripeness	Slope	0.0003	−0.0610	0.0010	0.0025	0.0027	−0.0036	0.0855	−0.0004
		Offset	4.97	10.83	3.63	1.35	1.16	1.26	718.57	0.78
		RMSE	473.25	469.23	474.18	475.76	475.89	475.82	516.79	476.16
		*R*^2^	0.00	0.00	0.00	0.02	0.07	0.01	0.04	0.00
N uptake	Anthesis	Slope	−0.0928	−0.9203	−0.0913	−0.0059	−0.0012	−0.0031	−0.0447	−0.0079
		Offset	6.51	23.60	5.19	1.46	1.20	1.25	720.21	0.92
		RMSE	5.32	12.67	4.95	5.31	5.43	5.41	684.30	5.57
		*R*^2^	0.13^*^	0.35^**^	0.16^*^	0.09	0.02	0.15^*^	0.02	0.23^**^
	Dough ripeness	Slope	0.0001	−0.0002	0.0001	0.0000	0.0000	0.0000	0.0010	0.0000
		Offset	5.10	11.26	3.71	1.35	1.16	1.28	718.58	0.80
		RMSE	4.00	8.24	3.69	4.30	4.40	4.34	776.37	4.61
		*R*^2^	0.00	0.00	0.00	0.02	0.11	0.05	0.05	0.00

Significant R^2^-values are indicated at

* p ≤ 0.05 and

** *p ≤ 0.01*.

### Detection of the dry weight and N uptake of culms

The descriptive statistics of the culms are given in Supplementary Table 3. The culm N uptake in 2013 and 2015 was on a comparable level; however, in 2014, 38% less nitrogen was accumulated at anthesis. In contrast to 2013 and 2015, in 2014, the culms reached the highest dry weight at dough ripeness. In the other years, decreasing dry weight in later growth stages was observed. For the plotwise PLSR (Table 10), good relationships with *R*^2^ = 0.53–0.66 were found for both the dry weight and N uptake of culms. However, the N uptake at anthesis showed a high number of PC and a marked difference between the calibration and validation results. The genotype-wise differentiation delivered comparable results.

**Table 10:** Results of the plotwise PLSR analysis of the dry weight and N uptake of culms used for calibration and validation; the genotype-wise differentiation is contained in Supplementary Tables 5, 6.

		**PC**	**Cal**				**Val**			
			**Slope**	**Offset**	**RMSE**	***R*^2^**	**Slope**	**Offset**	**RMSE**	***R*^2^**
Anthesis	DW culms (kg/ha)	5	0.59	1627	721.76	0.59	0.63	1470	728.36	0.54
	N uptake culms (kg/ha)	7	0.66	23.64	12.41	0.66	0.73	19.77	12.31	0.53
Dough ripeness	DW culms (kg/ha)	4	0.65	1317	606.73	0.65	0.54	1635	763.03	0.61
	N uptake culms (kg/ha)	6	0.61	6.1	4.45	0.61	0.55	7.0	4.72	0.60

The results of the linear regressions are shown in Table 11. The passive spectrometer showed an improved performance with regard to the R900/R970 and R780/R670 indices, whereas no further improvement was obtained from the Contour Map analysis. The coefficients of determination of the VIs and PLSR were comparable; however, the RMSEs for the VIs were almost four times higher. The best performance of the passive spectrometer was obtained at dough ripeness, whereas the CropCircle showed enhanced performance at anthesis.

**Table 11:** Results of linear regression analysis of the dry weight and N uptake of culms, showing the tested vegetation indices from three active and one passive spectral sensor at anthesis and dough ripeness.

**Culms in (kg/ha)**			**CropCircle**				**GreenSeeker**		**AFS**	
			**R730/R670**	**R760/R730**	**R760/R670**	**NDVI**	**R774/R656**	**NDVI**	**R760/R730**	**R900/R970**
Dry weight	Anthesis	Slope	0.0001	0.0001	0.0007	0.0001	0.0003	0.0000	0.0000	0.0000
		Offset	0.83	1.12	−0.98	0.09	1.99	0.32	1.45	0.94
		RMSE	4,476.54	4,476.26	4,478.30	4,477.26	4,179.85	4,181.47	4,180.37	4,180.87
		*R*^2^	0.49^**^	0.56^**^	0.64^**^	0.59^**^	0.11	0.14^*^	0.02	0.33^**^
	Dough ripeness	Slope	0.0003	0.0001	0.0009	0.0001	0.0004	0.0001	0.0001	0.0000
		Offset	0.50	1.08	−1.23	−0.06	1.08	0.15	1.19	0.97
		RMSE	3,953.33	3,952.78	3,954.98	3,953.86	3,872.40	3,873.29	3,872.30	3,872.51
		*R*^2^	0.46^**^	0.29^**^	0.37^**^	0.39^**^	0.34^**^	0.34^**^	0.43^**^	0.08
N uptake	Anthesis	Slope	0.0051	0.0023	0.0092	0.0019	−0.0196	−0.0023	−0.0033	−0.0003
		Offset	0.99	1.47	1.14	0.23	4.59	0.64	1.76	1.04
		RMSE	66.23	65.77	66.07	66.95	68.07	71.78	70.72	71.40
		*R*^2^	0.13	0.03	0.02	0.08	0.15^*^	0.16^*^	0.18^*^	0.03
	Dough ripeness	Slope	0.0531	0.0294	0.1783	0.0244	0.0579	0.0094	0.0099	0.0009
		Offset	0.66	1.16	−0.64	0.01	1.75	0.26	1.28	1.00
		RMSE	17.74	17.76	17.57	17.78	17.47	17.62	17.61	17.62
		*R*^2^	0.37^**^	0.25^**^	0.29^**^	0.34^**^	0.24^**^	0.24^**^	0.38^**^	0.02
**Culms (kg/ha)**			**Passive spectrometer**							
			**R780/R550**	**R780/R670**	**R780/R700**	**R760/R730**	**R780/R740**	**R900/R970**	**REIP**	**NDVI**
Dry weight	Anthesis	Slope	0.0004	0.0016	0.0004	0.0000	0.0000	0.0000	0.0006	0.0000
		Offset	3.92	6.59	2.31	1.22	1.16	1.16	716.82	0.67
		RMSE	4,177.99	4,175.40	4,179.54	4,180.60	4,180.66	4,180.66	3,495.44	4,181.13
		*R*^2^	0.10	0.05	0.14	0.16^*^	0.05	0.13	0.15^*^	0.14
	Dough ripeness	Slope	0.0006	0.0029	0.0006	0.0001	0.0000	0.0000	0.0012	0.0001
		Offset	2.07	−2.56	0.81	1.07	1.13	1.04	713.18	0.37
		RMSE	3,871.46	3,875.85	3,872.66	3,872.41	3,872.36	3,872.44	3,200.45	3,873.08
		*R*^2^	0.46^**^	0.56^**^	0.50^**^	0.47^**^	0.21^**^	0.56^**^	0.27^**^	0.49^**^
N uptake	Anthesis	Slope	−0.0126	−0.1624	−0.0179	−0.0009	0.0003	−0.0004	−0.0006	−0.0016
		Offset	6.21	24.14	5.15	1.43	1.17	1.23	719.41	0.91
		RMSE	66.53	51.47	67.54	71.03	71.28	71.22	649.93	71.52
		*R*^2^	0.04	0.18^*^	0.09	0.03	0.03	0.03	0.00	0.10
	Dough ripeness	Slope	0.0940	0.4410	0.0923	0.0095	0.0017	0.0070	0.1788	0.0132
		Offset	2.96	1.16	1.63	1.16	1.14	1.10	714.86	0.49
		RMSE	17.22	13.82	17.34	17.61	17.62	17.62	112.55	17.61
		*R*^2^	0.38^**^	0.49^**^	0.43^**^	0.39^**^	0.16^*^	0.50^**^	0.22^**^	0.42^**^

Significant R^2^-values are indicated at

* p ≤ 0.05 and

** *p ≤ 0.01*.

### Detection of the dry weight and N uptake of ears

The lowest dry weights and N uptake were observed in 2014 (Supplementary Table 4). The results of the plotwise PLSR analysis are shown in Table 12. Comparable results were provided by the genotype-wise differentiation as shown in the Supplementary Tables 5, 6. Medium correlations between the biomass parameters and wavelengths were found. Additionally, a high number of PC was found to be optimal.

**Table 12:** Results of the plotwise PLSR analysis of the dry weight and N uptake of ears used for calibration and validation; the genotype-wise differentiation is contained in Supplementary Tables 5, 6.

		**PC**	**Cal**				**Val**			
			**Slope**	**Offset**	**RMSE**	***R*^2^**	**Slope**	**Offset**	**RMSE**	***R*^2^**
Dough ripeness	DW ears (kg/ha)	7	0.57	2,299	924.41	0.57	0.64	2,158	951.59	0.49
	N uptake ears (kg/ha)	7	0.57	29.7	12.19	0.57	0.63	27.8	12.46	0.50

Considering the linear regressions of the vegetation indices shown in Table 13, no correlations were observed. The RMSEs were as high as the mean values observed for the biomass parameters. The Contour Map analysis did not lead to improved results (data not shown).

**Table 13:** Results of linear regression analysis of the dry weight and N uptake of ears, showing the tested vegetation indices from three active and one passive spectral sensor at dough ripeness.

**Ears (kg/ha)**		**CropCircle**				**GreenSeeker**		**AFS**	
		**R730/R670**	**R760/R730**	**R760/R670**	**NDVI**	**R774/R656**	**NDVI**	**R760/R730**	**R900/R970**
Dry weight	Slope	0.0001	0.0001	0.0003	0.0000	0.0001	0.0000	0.0000	0.0000
	Offset	0.79	1.23	0.15	0.09	2.41	0.40	1.50	0.98
	RMSE	5,475.74	5,475.32	5,476.36	5,476.41	5,478.77	5,480.70	5,479.64	5,480.14
	*R*^2^	0.10	0.07	0.05	0.08	0.01	0.00	0.02	0.03
N uptake	Slope	0.0072	0.0034	0.0189	0.0028	0.0045	0.0002	−0.0011	0.0004
	Offset	0.79	1.27	0.18	0.10	2.36	0.40	1.51	0.98
	RMSE	70.58	70.13	71.14	71.24	69.19	71.09	70.02	70.53
	*R*^2^	0.11	0.05	0.05	0.07	0.01	0.00	0.03	0.03
**Ears (kg/ha)**		**Passive spectrometer**							
		**R780/R550**	**R780/R670**	**R780/R700**	**R760/R730**	**R780/R740**	**R900/R970**	**REIP**	**NDVI**
Dry weight	Slope	0.0000	−0.0004	−0.0001	0.0000	0.0000	0.0000	0.0000	0.0000
	Offset	4.72	10.23	3.52	1.33	1.14	1.27	717.92	0.78
	RMSE	5476.54	5471.22	5477.69	5479.81	5479.99	5479.87	4791.26	5480.34
	*R*^2^	0.00	0.01	0.01	0.00	0.07	0.04	0.00	0.02
N uptake	Slope	−0.0051	−0.0296	−0.0064	−0.0004	0.0004	−0.0009	−0.0079	−0.0013
	Offset	4.81	10.26	3.55	1.33	1.14	1.27	718.26	0.79
	RMSE	66.88	61.88	68.08	70.19	70.37	70.25	644.12	70.72
	*R*^2^	0.01	0.01	0.01	0.00	0.06	0.05	0.00	0.03

Significant R^2^-values are indicated at ^*^p ≤ 0.05 and ^**^p ≤ 0.01.

### Detection of the number of ears per square meter

Low coefficients of determination were found by the plotwise PLSR analysis (Table 14). The lowest number of ears per area was consistently found for the hull-less barley cultivars with 200–300 ears per square meter in 2013 and 2015. Furthermore, the linear regressions between the ears per square meter and the vegetation indices indicated much lower relationships (Table 15) than the PLSR while showing very large RMSEs.

**Table 14:** Results of the plotwise PLSR analysis of the number of ears/sqm used for calibration and validation; the genotype-wise differentiation is contained in Supplementary Tables 5, 6.

**Ears sqm^−1^**	**PC**	**Cal**				**Val**			
		**Slope**	**Offset**	**RMSE**	***R*^2^**	**Slope**	**Offset**	**RMSE**	***R*^2^**
Dough ripeness	5	0.38	394.2	91.5	0.38	0.35	429	91.3	0.31

**Table 15:** Results of linear regression analysis of the number of ears per square meter, showing the tested vegetation indices from three active and one passive spectral sensor at dough ripeness.

**Ears/sqm**		**CropCircle**				**GreenSeeker**		**AFS**	
		**R730/R670**	**R760/R730**	**R760/R670**	**NDVI**	**R774/R656**	**NDVI**	**R760/R730**	**R900/R970**
Dough ripeness	Slope	0.0010	0.0005	0.0028	0.0004	0.0022	0.0002	0.0002	0.0000
	Offset	0.69	1.19	−0.17	0.04	1.23	0.21	1.24	0.96
	RMSE	655.42	654.92	656.26	656.05	655.16	656.17	655.15	655.43
	*R*^2^	0.13	0.08	0.07	0.10	0.09	0.06	0.08	0.03
**Ears/sqm**		**Passive spectrometer**							
		**R780/R550**	**R780/R670**	**R780/R700**	**R760/R730**	**R780/R740**	**R900/R970**	**REIP**	**NDVI**
Dough ripeness	Slope	0.0032	0.0129	0.0026	0.0003	0.0001	0.0001	0.0059	0.0003
	Offset	2.37	−0.15	1.36	1.10	1.09	1.09	713.87	0.46
	RMSE	654.03	656.42	655.03	655.29	655.31	655.31	136.46	655.93
	*R*^2^	0.11	0.11	0.09	0.11	0.20	0.08	0.06	0.08

Significant R^2^-values are indicated at ^*^p ≤ 0.05 and ^**^p ≤ 0.01.

### Predictive performance of organ-specific barley PLSR models

The barley PLSR models were used to predict the organ-specific assessment of an independent set of 13 wheat cultivars grown in the years 2015 and 2016. Dry weights of wheat leaves were predicted with Pearson correlation coefficients (*R*^2^-values) of 0.73 and 0.52 in 2015 and 2016, respectively, total plant dry weight with *R*^2^-values of 0.45 and 0.52, and culm dry weights with *R*^2^-values of 0.23 and 0.32 (Lukas Prey, personal communication 2017). N uptake of the total biomass was predicted with *R*^2^-values of 0.70 and 0.57 in 2015 and 2016, respectively, N uptake of leaves with *R*^2^ = 0.64 and 0.70, and N uptake of culms with *R*^2^ = 0.70 and 0.44, for 2015 and 2016, respectively.

## Discussion

The performance of three active spectrometers and one passive spectrometer was evaluated to detect differences in the measured dry weight and nitrogen uptake of leaves, leaf sheaths, culms, and ears of a set of 30–34 spring barley cultivars at anthesis and dough ripeness. Furthermore, contour maps and PLSR were compared with various published vegetation indices.

### Published vegetation indices

Considering the performance of the published VIs, the index R780/R670 was found to be most closely related to the biomass parameters of leaves and culms. Saturation effects of the NDVI were observed, especially for the passive spectrometer and the GreenSeeker at anthesis. The same problem was reported by Haboudane et al. (2004). In general, moderate coefficients of determination were observed between the published VIs and the dry weight and nitrogen uptake of culms and leaves. Other studies on spring barley (e.g., Behrens et al., 2006; Bendig et al., 2015; Elsayed et al., 2015; Tilly et al., 2015) presented better or at least similar results; however, those previous findings were based on different fertilizer levels or varying levels of drought and heat stress.

Although Erdle et al. (2013) reported that the R760/R730 index is suitable for the detection of the dry weight of ears in winter wheat, neither a published VI nor the PLSR was able to provide satisfactory relationships for spring barley. The Nadir positioning of the sensors could be a possible reason. Since the ears were still in a vertical posture at dough ripeness, the sensors may not have been able to detect these organs.

### Contour map analysis

The contour map analysis, testing all possible dual indices, did not provide improved results compared to the selected indices. While Li et al. (2013a,b), Elsayed et al. (2015), Rischbeck et al. (2016) and Yu et al. (2012) indicated improvements of contour map based vegetation indices compared to published VIs, no improved wavelength combination was found in this study. Although Elsayed et al. (2015) and Rischbeck et al. (2016) used a similar set of cultivars and the same sensors, their results differ from those found in this study. This discrepancy might be due to the increased variance in their studies induced by different nitrogen fertilizer levels or drought stress levels.

### PLSR

In general, the plotwise and the genotype-wise PLSR analysis of the dry weight and N uptake of the different organs (ears, leaves, culms) as well as of the total biomass delivered comparable results at anthesis (Tables 6, 8, 10, 12, 14 and Supplementary Tables 5, 6, respectively). Partly the genotype-wise calibration models delivered even slightly improved relationships compared to the plotwise calibration models, whereas this was less manifest for the validation models. No satisfactory results were achieved for the genotype-wise calibration and validation models for the N-uptake of leaf sheaths.

At dough ripeness quite comparable results were observed for the organ-specific differentiation of the plot-wise and genotype-wise PLSR analysis for the calibration models, whereas the plotwise validation models were less good. No satisfactory results were obtained for the N uptake of leaf sheaths.

Without exception, PLSR analysis outperformed the simple vegetation indices as well as the indices derived from contour map analysis. Markedly reduced RMSEs and higher coefficients of determination were achieved by PLSR, in agreement with the results from other studies on winter wheat (Hansen and Schjoerring, 2003; Li et al., 2013a), spring wheat (Øvergaard et al., 2013), durum wheat (Ferrio et al., 2005) and spring barley (Elsayed et al., 2015). Øvergaard et al. (2013) reported that at least 2 years of data are necessary to obtain stable PLSR models. The results from this study are in line with this recommendation since the PLSR models showed increased precision when further data were added (results not shown). The best results were obtained for leaves, culms and leaf sheaths at anthesis. However, for culms at anthesis and ears, a marked difference between the calibration and validation models was obtained. A large number of principle components points to a rather unstable model.

At anthesis the main spectral wavelengths, disregarding the signs of the correlation coefficients, contributing to the assessment of the dry weights of culms and the total dry weight were found in the waveband regions of 930–999 and 529–558 nm, whereas the leaf dry weight was more specifically related to wavebands at 996–999, 948, and 961, 890–929, and 725–738 nm (Supplementary Table 7). N uptake of culms and leaves was most closely related to spectral information found between 526 and 591 nm, with the N uptake of culms being further related to the spectral regions at 930–998 nm and at 865–890 nm.

At dough ripeness partly comparable regions were identified being related to the dry weights of culms and the total plant, however being more specifically located at 996–999 nm for the culms and at 935–948 nm for the total dry weight (Supplementary Table 8). In accordance with anthesis spectral information at 529–568 nm was related to the dry weights of culms and the total plant at dough ripeness and further useful information was found at 653–686 nm and at 715–725 nm. A close agreement regarding the spectral regions related to the leaf dry weight was evident at dough ripeness compared to anthesis. N uptake of culms was most closely related to information found at 699–722 nm, but was also related to information found at 530–568 and 660–683 nm. N uptake of leaves was most closely related to information found at 702–722 nm as well to 401–509 nm. N uptake of the total aboveground biomass was best related to information found in the 894–929 nm region, as well to wavebands at 932, 958, 974 nm, and additionally to information found in the spectral regions between 575–598 nm and at 532–545 nm. Ear dry weights were best reflected by spectral information found at 709–732 nm and at 929–948 nm as well as at 999 nm, whereas the N uptake of the ears was best related to the spectral information found at 663–686, 706–732, and 929–948 nm.

### Comparison of sensors

Several comparisons between spectral proximal sensors have been previously performed. Erdle et al. (2011), Winterhalter et al. (2013), and Elsayed et al. (2015) found a slight advantage of the passive spectrometer, in particular, when nitrogen parameters were detected. These findings were confirmed by this study. The R780/R670 index and NDVI were revealed to be more precise when measured with the passive spectrometer. The performance of the active sensors depends on their light source, which is weaker than sunlight (Winterhalter et al., 2013). Furthermore, their performance depends on the target distance. The emitted light follows the inverse square law. A doubled measuring distance leads to a four times lower light intensity (Kipp et al., 2014). Since the sensor carrier was positioned 1 m above the plant canopy (in line with the recommendations of the manufacturers), differences in the canopy density, plant architecture and penetration depth may contribute to the slightly decreased performance of the active sensors (Winterhalter et al., 2013; Kipp et al., 2014).

### Biomass parameters

The year 2013 was characterized by remarkably low heritability of all plant organs (*h*^2^ = 0.18–0.49) due to severe weather conditions and a flood in certain areas of the field trial. This also led to an inconsistent dry weight and N uptake of the cultivars. The highest and most consistent heritability was observed for leaves (*h*^2^ = 0.75–0.85), whereas culms showed a low heritability (*h*^2^ = 0.31–0.38), particularly at dough ripeness.

The dry weight and N uptake of leaves are important factors that plant breeders use to assess the photosynthetic potential of a plant (Zhu et al., 2010; Acquaah, 2012). In this study, the dry weight of leaves amounted to 25% of the total aboveground biomass and accumulated up to 30% of the total N uptake at anthesis.

The dry weight of culms was ~75% of the total aboveground biomass and stored ~70 kg N ha^−1^ at anthesis. At dough ripeness, only 16 kg N ha^−1^ remained within the culm biomass. These findings are in line with the studies of Bidinger et al. (1977) and Mirosavljevic et al. (2015), which described the culm as the most important storage organ.

The leaf sheaths showed inconsistent behavior. While culms and leaves translocated dry weight and nitrogen during grain filling, leaf sheaths accumulated dry weight and nitrogen. The assumption of Schnyder (1993), who identified wheat leaf sheaths as a type of storage organ, were supported in this study for barley. Furthermore, the spectral sensors showed limitations considering the detection of leaf sheaths, especially at dough ripeness. In this growth stage, only a weak relationship (*R*^2^ = 0.27) between the total aboveground biomass and leaf sheaths was found, and no relationships were observed between the biomass parameters of leaf sheaths and leaves or culms.

The same results were obtained for the relationships of the biomass parameters of ears with the other plant organs. However, a highly significant relationship (*R*^2^ = 0.73) was found for the dry weight of ears at dough ripeness and total biomass at anthesis.

It is assumed that the detectability of different plant organs is mainly influenced by their contribution to the total aboveground biomass. Furthermore, a correlation analysis revealed fair relationships between the total dry weight and good relationships (*R*^2^ = 0.70) between the number of ears sqm^−1^ and the final grain yield. However, the PLSR analysis of the number of ears sqm^−1^ indicated low coefficients of determination and it is concluded, that the detectability of the ears and the number of ears is hardly possible by spectral proximal sensing. The general detectability of the plant organs can be described as leaves>culms>ears>leaf sheaths, while anthesis>dough ripeness and N uptake>dry weight. Considering the analysis of the data, an enhanced performance of PLSR compared to vegetation indices was observed. The quality of the sensors is mainly influenced by their light source and was found as: passive bidirectional spectrometer ≥ CropCircle > GreenSeeker = AFS.

Nevertheless, spectral proximal sensing combined with suitable PLSR models maybe a convenient method for obtaining information about leaves and culms at anthesis and dough ripeness. The PLS method revealed to be a rather good postdictive method, better than any single spectral index. This is corroborated by a recent study where yield and protein content prediction in independent field studies was successfully demonstrated (Barmeier et al., 2017). This method delivered improved results compared to optimized spectral indices to estimate the nitrogen content (Li et al., 2013b) or the yield of wheat (Becker and Schmidhalter, 2017), and allowed by to predict drought stress and grain yield in barley (Elsayed et al., 2015; Rischbeck et al., 2016).

The predictive performance of the organ-specific barley models was tested to predict organ-specific information of a set of wheat cultivars grown in two years, which allowed for an independent testing of the barley PLSR models. Interestingly enough a fairly good prediction of wheat organ-specific information was obtained at anthesis for the dry weight of leaves, a moderate for the dry weight of the total plant, whereas a decreased performance was obtained for the culm dry weight. Regarding the nitrogen related parameters, a good prediction was obtained for the N uptake of leaves, as well as rather close relationships were obtained for the N uptake of the total biomass, and a fairly good prediction was observed for the N uptake of culms. Seen that a model which had been optimized for barley plants could be transferred to wheat plants, this is an encouraging result, which should further be substantiated. To the best of our knowledge this is the first report showing an independent calibration and validation for organ-specific information of barley and wheat plants and the possible transfer of models. In view of the marked differences in the growth habit of these two species, this represents an interesting observation, pointing to some commonalities in the spectral information. Different models which were obtained for different years further indicates, that a relative classification of the performance of cultivars seems to be possible. It has previously been shown that also vegetation indices do not predict absolute differences, but allow for a relative year-specific differentiation (Hackl et al., 2013). Still, a relative differentiation or robust sculling of better performing genotypes at given times is highly useful, being congruent with the relative scoring adopted by breeders.

Spectral information being more closely related to the specific organs was identified. This seems to vary depending on the organ, even though some commonalities could also be observed, with interesting observation being detected in the photosynthetic active radiation range (495–700 nm), but also being contained in the red edge region extending to the near infrared regions (680–780 nm) and the water index region and slightly beyond it. Reflectance measurements in these ranges might be of use to measure these organs and incorporate them as novel selection criteria to optimize the architecture of plants and for improving yield related parameters. At present spectral indices allowing to identify organ-specific information may lend for a quicker adoption in field experimentation and plant breeding. There is a need for further studies to confirm the PLSR results by testing spectral indices that have consistently large loadings in the PC and to assess their predictive validity.

A suitable phenotyping platform enhances the performance of phenotyping. By driving at an average speed of ~5.5 km h^−1^, the measurement of a single plot takes ~0.8–1.8 s, while destructive measurements with subsequent laboratory analysis is tedious and time consuming. Spectral sensors are non-invasive and objective and therefore offer an enhanced tool that can keep pace with high-throughput genotyping techniques and thereby widen the phenotyping bottleneck (Winterhalter et al., 2011; White et al., 2012; Kipp et al., 2014; Becker and Schmidhalter, 2017).

Our results may lead to a better understanding of the information gained from spectral measurements of plant canopies, thereby being of potential use for enhanced phenotyping and architectural modeling. A better understanding of the plant architecture may allow for a more targeted breeding (Winterhalter et al., 2012). Organ specific phenotyping represents a first possible step toward breeding by design.

## Author contributions

GB and US conceived and designed the experiments and wrote the paper; GB performed the experiments and analyzed the data.

### Conflict of interest statement

The authors declare that the research was conducted in the absence of any commercial or financial relationships that could be construed as a potential conflict of interest.
