# Supplementary material for: High-Throughput Field Phenotyping of Leaves, Leaf Sheaths, Culms and Ears of Spring Barley Cultivars at Anthesis and Dough Ripeness
Source: Front Plant Sci. 2017 Nov 7;8:1920. doi: 10.3389/fpls.2017.01920 (PMC5681945; doi:10.3389/fpls.2017.01920)
Supplement: Supplementary file 1 [file Table1.PDF]

Supplementary Material

Active and passive high-throughput field phenotyping of leaves, leaf sheaths, culms and ears of spring barley cultivars at anthesis and dough ripeness

Gero Barmeier[1] and Urs Schmidhalter[1]*

[1]Chair of Plant Nutrition, Department of Plant Sciences, Technical University of Munich, Emil-Ramann-Str. 2, 85354 Freising, Germany

*Corresponding author: Tel.: +0049 8161 713390; Fax: +0049 8161 714500;
E-mail: schmidhalter@wzw.tum.de

Supplementary Table 1 Descriptive statistics of barley leaves

	LEAVES	Anthesis		Dough ripeness	
Year		Dry weight leaves in kg ha^{-1}	N uptake leaves in kg ha^{-1}	Dry weight leaves in kg ha^{-1}	N uptake leaves in kg ha^{-1}
2015	CV (%)	22.41	25.06	23.95	30.52
	Heritability (h²)	0.75	0.84	0.74	0.84
	Std. error	21.86	0.65	20.64	0.44
	Std. Dev.	245.36	7.33	228.95	4.79
	N	126	126	123	121
	Min	397.93	8.11	327.82	3.07
	Max	1680.26	44.80	1548.66	27.81
	Mean	918.65	24.57	818.98	12.40
2014	CV (%)	14.90	20.35	18.53	26.74
	Heritability (h²)	0.69	0.66	0.71	0.71
	Std. error	28.08	0.79	27.43	0.55
	Std. Dev.	297.20	8.33	289.03	5.83
	N	112	111	111	111
	Min	629.18	15.50	515.40	5.89
	Max	2451.52	55.77	1904.66	29.79
	Mean	1271.08	30.27	1052.43	15.39
2013	CV (%)	18.42	26.74	17.63	23.46
	Heritability (h²)	0.38	0.49	0.30	0.24
	Std. error	15.65	0.31	12.93	0.17
	Std. Dev.	167.82	3.31	138.10	1.76
	N	115	115	114	113
	Min	334.49	4.05	306.62	2.09
	Max	1277.08	22.01	1160.99	12.41

	Mean	815.20	10.56	772.71	7.09

Supplementary Table 2 Descriptive statistics of barley leaf sheaths

	LEAF SHEATHS	Anthesis		Dough ripeness	
Year		Dry weight leaf sheaths in kg ha^{-1}	N uptake leaf sheaths in kg ha^{-1}	Dry weight leaf sheaths in kg ha^{-1}	N uptake leaf sheaths in kg ha^{-1}
2015	CV (%)	21.14	21.24	34.39	27.68
	Heritability (h^2)	0.39	0.78	0.53	0.67
	Std. error	15.43	0.27	28.10	0.31
	Std. Dev.	173.23	3.06	311.65	3.36
	N	126	126	123	120
	Min	315.29	4.82	286.62	3.97
	Max	1189.40	22.30	1943.35	23.65
	Mean	698.55	11.99	866.71	10.63
2014	CV (%)	35.35	45.97	16.86	20.10
	Heritability (h^2)	0.27	0.30	0.46	0.53
	Std. error	15.43	0.19	13.13	0.15
	Std. Dev.	163.28	1.97	138.34	1.60
	N	112	111	111	111
	Min	240.23	1.96	344.96	3.58
	Max	1698.50	19.70	984.95	11.07
	Mean	425.65	4.02	640.87	6.77

Supplementary Table 3 Descriptive statistics of barley culms

	CULMS	Anthesis		Dough ripeness	
Year		Dry weight culms in kg ha^{-1}	N uptake culms in kg ha^{-1}	Dry weight culms in kg ha^{-1}	N uptake culms in kg ha^{-1}
2015	CV (%)	17.87	19.12	21.36	24.74
	Heritability (h^2)	0.57	0.73	0.31	0.38
	Std. error	94.00	1.61	81.82	0.36
	Std. Dev.	1055.11	18.08	907.38	4.02
	N	126	126	123	122
	Min	2463.03	29.34	1701.83	5.59
	Max	7622.66	135.50	6533.70	29.70
	Mean	4842.49	77.07	3631.65	14.31
2014	CV (%)	15.49	17.25	15.96	25.71
	Heritability (h^2)	0.77	0.68	0.68	0.55
	Std. error	82.64	1.07	102.39	0.77
	Std. Dev.	874.56	11.23	1078.79	8.12
	N	112	111	111	111
	Min	1855.09	26.84	2513.00	11.40
	Max	6896.53	86.21	8365.79	51.42
	Mean	3820.60	49.87	4710.70	22.94
2013	CV (%)	20.94	17.74	19.37	21.54
	Heritability (h^2)	0.37	0.14	0.38	0.38
	Std. error	65.70	1.38	50.50	0.26
	Std. Dev.	704.51	14.80	539.19	2.77
	N	115	115	114	114
	Min	1393.72	30.87	1435.54	5.57
	Max	5522.94	119.34	4396.35	18.42

	Mean	3259.61	80.92	2790.98	11.21

Supplementary Table 4 Descriptive statistics of barley ears

	EARS	Dough ripeness	
Year		Dry weight ears in kg ha^{-1}	N uptake ears in kg ha^{-1}
2015	CV (%)	16.95	18.71
	Heritability (h²)	0.67	0.78
	Std. error	111.12	1.53
	Std. Dev.	1227.37	16.81
	N	122	120
	Min	2632.56	35.36
	Max	9479.07	123.13
	Mean	5936.00	77.83
2014	CV (%)	20.36	21.27
	Heritability (h²)	0.63	0.66
	Std. error	108.79	1.39
	Std. Dev.	1146.17	14.65
	N	111	111
	Min	1877.72	27.07
	Max	7379.50	107.15
	Mean	4200.37	54.56
2013	CV (%)	16.63	17.45
	Heritability (h²)	0.18	0.28
	Std. error	94.17	1.22
	Std. Dev.	1005.45	13.03
	N	114	114
	Min	2132.40	30.66
	Max	8179.26	103.44
	Mean	5816.68	76.11

Abbreviations of the ANOVA dataset

**Abbreviation**	**Plant organ/Sensor**	**Vegetation Index**
**DWears**	Dry weight ears in kg/ha	
**DWleaves**	Dry weight leaves in kg/ha	
**DWsheaths**	Dry weight leaf sheaths in kg/ha	
**DWculms**	Dry weight culms in kg/ha	
**DWtotal**	Dry weight total biomass in kg/ha	
**NupEars**	N uptake ears in kg/ha	
**NupLeaves**	N uptake leaves in kg/ha	
**NupSheaths**	N uptake leaf sheaths in kg/ha	
**NupCulms**	N uptake culms in kg/ha	
**NupTotal**	N uptake total biomass in kg/ha	
**CC730_670**	CropCircle	R730/R670
**CC760_730**	CropCircle	R760/R730
**CC760_670**	CropCircle	R760/R670
**CCNDVI**	CropCircle	NDVI
**GS774_656**	GreenSeeker	R774/R656
**GSNDVI**	GreenSeeker	NDVI
**ALS760_730**	Yara ALS(AFS)	R760/R730
**ALS900_970**	Yara ALS(AFS)	R900/R970
**PS780_550**	Passive spectrometer	R780/R550
**PS780_670**	Passive spectrometer	R780/R670
**PS780_700**	Passive spectrometer	R780/R700
**PS760_730**	Passive spectrometer	R760/R730
**PS780_740**	Passive spectrometer	R780/R740
**PS900_970**	Passive spectrometer	R900/R970
**PSREIP**	Passive spectrometer	REIP
**PSNDVI**	Passive spectrometer	NDVI

Supplementary Table 5 Genotype-wise PLSR of dry weights and N uptake of different plant organs at anthesis

**ANTHESIS**	**Cal**					**Val**			
**PLSR**	**PC**	**Slope**	**Offset**	**RMSE**	**R^2**	**Slope**	**Offset**	**RMSE**	**R^2**
**DW Leaves (kg/ha)**	2	0.58	400.8	117.28	0.58	0.48	501.6	136.3	0.50
**DW Leaf sheaths (kg/ha)**	4	0.45	309.20	56.65	0.45	0.24	433.80	75.53	0.12
**DW Culms (kg/ha)**	5	0.73	1039.40	265.35	0.73	0.51	1942.20	375.55	0.46
**DW Total biomass (kg/ha)**	5	0.80	1007.5	320.67	0.80	0.64	1903.10	448.72	0.64
**N-uptake Leaves (kg/ha)**	3	0.72	5.72	2.58	0.72	0.66	6.90	3.06	0.66
**N-uptake Leaf sheaths (kg/ha)**	1	0.01	8.18	1.27	0.01	0.00	9.10	1.41	0.00
**N-uptake Culms (kg/ha)**	5	0.68	21.80	3.99	0.68	0.62	25.80	4.95	0.46
**N-uptake Total biomass (kg/ha)**	2	0.65	32.90	6.00	0.65	0.52	45.40	7.06	0.57

Supplementary Table 6 Genotype-wise PLSR of dry weights and N uptake of different plant organs at dough ripeness

**DOUGH RIPENESS**	**Cal**					**Val**			
**PLSR**	**PC**	**Slope**	**Offset**	**RMSE**	**R^2**	**Slope**	**Offset**	**RMSE**	**R^2**
**DW Ears (kg/ha)**	4	0.67	1681.2	414.51	0.67	0.45	2909.9	612.89	0.32
**DW Leaves (kg/ha)**	5	0.75	207.90	74.55	0.75	0.50	418.50	121.52	0.41
**DW Leaf sheaths (kg/ha)**	1	0.01	739.10	108.02	0.01	0.00	807.00	118.32	0.00
**DW Culms (kg/ha)**	2	0.56	1551.1	381.05	0.56	0.54	1636.40	429.72	0.49
**DW Total biomass (kg/ha)**	2	0.4	6066.30	874.57	0.40	0.33	6740.60	1035.20	0.21
**N-uptake Ears (kg/ha)**	4	0.57	29.40	4.78	0.57	0.35	45.00	6.63	0.23
**N-uptake Leaves (kg/ha)**	2	0.54	5.17	1.80	0.54	0.50	5.45	2.14	0.43
**N-uptake Leaf sheaths (kg/ha)**	1	0.02	8.36	1.17	0.02	0.00	9.00	1.27	0.00
**N-uptake Culms (kg/ha)**	5	0.8	3.08	1.71	0.80	0.68	4.88	2.74	0.48
**N-uptake Total biomass (kg/ha)**	2	0.44	55.24	7.65	0.44	0.39	60.22	9.03	0.23
**Ears/sqm**	1	0.08	572.40	87.81	0.08	0.00	623.90	99.01	0.00

Supplementary Table 7 Important wavelengths according the PLSR analysis at anthesis. Red = high influence; Green = low influence

Wavelength	DW leaves	DW leaf sheaths	DW culm	DW total	N uptake leaves	N uptake leaf sheaths	N uptake culm	N uptake total
**401**	0.05	0.10	0.03	0.03	0.03	0.04	0.04	0.04
**404**	0.05	0.10	0.03	0.03	0.03	0.04	0.04	0.04
**408**	0.04	0.10	0.02	0.03	0.02	0.04	0.03	0.04
**411**	0.04	0.10	0.02	0.02	0.02	0.04	0.03	0.03
**414**	0.03	0.10	0.02	0.02	0.02	0.05	0.02	0.03
**418**	0.03	0.09	0.02	0.02	0.01	0.05	0.02	0.02
**421**	0.02	0.09	0.01	0.01	0.01	0.05	0.00	0.01
**424**	0.02	0.09	0.01	0.00	0.00	0.05	0.00	0.00
**427**	0.01	0.09	0.00	0.00	0.00	0.05	0.01	0.01
**431**	0.00	0.08	0.01	0.01	0.01	0.04	0.02	0.02
**434**	0.00	0.08	0.01	0.02	0.01	0.04	0.03	0.03
**437**	0.01	0.08	0.02	0.02	0.02	0.04	0.03	0.03
**441**	0.00	0.08	0.02	0.02	0.01	0.04	0.03	0.02
**444**	0.00	0.07	0.03	0.03	0.01	0.04	0.02	0.02
**447**	0.01	0.07	0.03	0.03	0.01	0.04	0.03	0.03
**450**	0.01	0.07	0.03	0.04	0.01	0.04	0.03	0.03
**454**	0.01	0.07	0.03	0.04	0.01	0.04	0.04	0.03
**457**	0.01	0.07	0.03	0.03	0.02	0.04	0.04	0.04
**460**	0.01	0.07	0.03	0.04	0.02	0.03	0.05	0.04
**464**	0.02	0.07	0.03	0.03	0.02	0.03	0.06	0.05
**467**	0.02	0.07	0.03	0.03	0.03	0.03	0.06	0.06
**470**	0.03	0.07	0.03	0.03	0.03	0.03	0.06	0.06
**473**	0.03	0.06	0.03	0.03	0.03	0.03	0.07	0.07
**477**	0.03	0.06	0.02	0.03	0.03	0.03	0.07	0.06
**480**	0.03	0.06	0.03	0.03	0.03	0.02	0.08	0.07
**483**	0.03	0.06	0.03	0.03	0.03	0.02	0.08	0.07
**486**	0.04	0.06	0.03	0.03	0.03	0.02	0.09	0.08
**490**	0.04	0.06	0.03	0.03	0.03	0.02	0.09	0.08
**493**	0.04	0.06	0.03	0.03	0.03	0.02	0.09	0.08
**496**	0.05	0.06	0.03	0.03	0.03	0.02	0.08	0.07
**500**	0.05	0.06	0.03	0.03	0.03	0.02	0.09	0.07
**503**	0.05	0.05	0.03	0.03	0.04	0.02	0.08	0.07
**506**	0.05	0.05	0.03	0.03	0.03	0.02	0.07	0.06
**509**	0.04	0.04	0.03	0.03	0.03	0.02	0.06	0.04
**513**	0.03	0.03	0.03	0.03	0.03	0.02	0.05	0.03

**516**	0.02	0.02	0.03	0.03	0.03	0.03	0.04	0.01
**519**	0.00	0.01	0.04	0.04	0.02	0.03	0.02	0.01
**523**	0.01	0.00	0.05	0.04	0.00	0.03	0.01	0.04
**526**	0.01	0.01	0.07	0.07	0.01	0.03	0.04	0.06
**529**	0.01	0.02	0.09	0.08	0.03	0.03	0.06	0.09
**532**	0.00	0.03	0.10	0.09	0.05	0.03	0.09	0.11
**536**	0.01	0.04	0.11	0.09	0.07	0.03	0.11	0.13
**539**	0.01	0.05	0.12	0.10	0.09	0.03	0.12	0.14
**542**	0.01	0.05	0.12	0.10	0.10	0.02	0.14	0.16
**546**	0.01	0.06	0.13	0.10	0.12	0.02	0.15	0.17
**549**	0.01	0.06	0.13	0.11	0.13	0.01	0.16	0.18
**552**	0.01	0.06	0.13	0.11	0.14	0.01	0.16	0.18
**555**	0.02	0.07	0.12	0.10	0.14	0.00	0.16	0.17
**559**	0.01	0.07	0.11	0.09	0.13	0.00	0.16	0.17
**562**	0.01	0.06	0.10	0.08	0.13	0.00	0.16	0.17
**565**	0.01	0.06	0.08	0.06	0.12	0.01	0.16	0.17
**569**	0.01	0.06	0.06	0.04	0.11	0.01	0.15	0.16
**572**	0.01	0.05	0.04	0.02	0.10	0.02	0.14	0.15
**575**	0.04	0.05	0.01	0.00	0.09	0.02	0.14	0.14
**578**	0.06	0.04	0.01	0.03	0.08	0.03	0.12	0.13
**582**	0.08	0.04	0.03	0.04	0.06	0.03	0.11	0.11
**585**	0.08	0.04	0.04	0.05	0.05	0.04	0.09	0.09
**588**	0.06	0.04	0.06	0.06	0.03	0.04	0.06	0.06
**592**	0.05	0.04	0.05	0.06	0.02	0.04	0.05	0.05
**595**	0.05	0.03	0.06	0.06	0.02	0.04	0.04	0.04
**598**	0.05	0.03	0.06	0.07	0.02	0.05	0.04	0.04
**601**	0.05	0.03	0.06	0.07	0.02	0.05	0.03	0.04
**605**	0.04	0.03	0.06	0.07	0.02	0.05	0.03	0.03
**608**	0.04	0.03	0.06	0.07	0.02	0.05	0.03	0.03
**611**	0.03	0.03	0.06	0.06	0.01	0.06	0.01	0.01
**615**	0.03	0.03	0.06	0.06	0.01	0.06	0.01	0.00
**618**	0.02	0.02	0.06	0.06	0.01	0.06	0.00	0.01
**621**	0.01	0.02	0.06	0.06	0.01	0.06	0.01	0.01
**624**	0.01	0.02	0.06	0.06	0.00	0.06	0.01	0.02
**628**	0.01	0.02	0.06	0.06	0.00	0.07	0.02	0.02
**631**	0.00	0.02	0.06	0.06	0.00	0.07	0.02	0.02
**634**	0.00	0.02	0.06	0.06	0.00	0.07	0.02	0.02
**637**	0.00	0.02	0.05	0.06	0.00	0.07	0.02	0.03
**641**	0.01	0.01	0.05	0.05	0.00	0.08	0.03	0.04
**644**	0.01	0.01	0.05	0.05	0.00	0.08	0.03	0.03
**647**	0.02	0.01	0.04	0.04	0.00	0.08	0.04	0.05
**651**	0.04	0.00	0.04	0.04	0.00	0.08	0.05	0.06
**654**	0.04	0.00	0.03	0.03	0.00	0.08	0.07	0.08

**657**	0.06	0.00	0.02	0.02	0.01	0.09	0.09	0.09
**660**	0.07	0.01	0.01	0.01	0.02	0.08	0.10	0.11
**664**	0.08	0.01	0.00	0.00	0.02	0.09	0.12	0.12
**667**	0.09	0.01	0.01	0.01	0.03	0.09	0.13	0.14
**670**	0.10	0.02	0.02	0.02	0.03	0.09	0.15	0.15
**673**	0.10	0.02	0.03	0.03	0.03	0.09	0.16	0.16
**677**	0.10	0.02	0.03	0.03	0.03	0.09	0.16	0.16
**680**	0.10	0.01	0.02	0.03	0.03	0.09	0.16	0.16
**683**	0.09	0.01	0.02	0.02	0.03	0.09	0.14	0.14
**687**	0.07	0.00	0.00	0.01	0.02	0.09	0.11	0.11
**690**	0.04	0.02	0.02	0.02	0.00	0.09	0.08	0.08
**693**	0.00	0.05	0.04	0.04	0.01	0.09	0.04	0.04
**696**	0.03	0.08	0.06	0.06	0.02	0.09	0.01	0.02
**700**	0.07	0.12	0.07	0.08	0.03	0.09	0.01	0.01
**703**	0.09	0.15	0.08	0.08	0.03	0.08	0.02	0.02
**706**	0.10	0.17	0.08	0.09	0.02	0.06	0.02	0.02
**709**	0.08	0.20	0.08	0.08	0.02	0.05	0.02	0.02
**713**	0.07	0.22	0.07	0.07	0.01	0.04	0.01	0.00
**716**	0.03	0.24	0.07	0.06	0.00	0.02	0.01	0.02
**719**	0.02	0.25	0.06	0.05	0.03	0.02	0.02	0.03
**722**	0.07	0.26	0.05	0.03	0.05	0.05	0.03	0.05
**726**	0.13	0.24	0.05	0.03	0.07	0.09	0.05	0.07
**729**	0.16	0.22	0.03	0.01	0.09	0.12	0.06	0.07
**732**	0.16	0.20	0.03	0.01	0.10	0.13	0.06	0.08
**736**	0.16	0.17	0.02	0.00	0.10	0.14	0.07	0.08
**739**	0.13	0.15	0.02	0.00	0.08	0.12	0.07	0.08
**742**	0.09	0.13	0.00	0.01	0.05	0.10	0.08	0.08
**745**	0.05	0.11	0.01	0.03	0.03	0.07	0.08	0.09
**749**	0.02	0.08	0.01	0.02	0.00	0.05	0.07	0.08
**752**	0.02	0.07	0.02	0.03	0.02	0.03	0.06	0.07
**755**	0.06	0.07	0.04	0.05	0.03	0.00	0.05	0.07
**758**	0.07	0.07	0.04	0.05	0.05	0.01	0.04	0.06
**762**	0.08	0.05	0.02	0.03	0.08	0.03	0.05	0.06
**765**	0.05	0.02	0.02	0.02	0.09	0.02	0.07	0.07
**768**	0.05	0.00	0.05	0.05	0.07	0.00	0.05	0.04
**771**	0.06	0.01	0.03	0.03	0.05	0.01	0.05	0.04
**775**	0.06	0.01	0.02	0.02	0.04	0.02	0.03	0.02
**778**	0.06	0.01	0.02	0.02	0.03	0.03	0.01	0.01
**781**	0.06	0.00	0.01	0.01	0.02	0.04	0.00	0.01
**784**	0.06	0.00	0.01	0.01	0.01	0.04	0.01	0.00
**788**	0.07	0.00	0.01	0.01	0.02	0.05	0.01	0.00
**791**	0.06	0.01	0.02	0.02	0.02	0.05	0.02	0.00
**794**	0.04	0.01	0.02	0.02	0.01	0.05	0.01	0.00
**797**	0.04	0.01	0.02	0.02	0.02	0.06	0.00	0.01

**801**	0.03	0.02	0.04	0.04	0.00	0.04	0.04	0.03
**804**	0.04	0.02	0.03	0.04	0.00	0.06	0.03	0.01
**807**	0.03	0.02	0.03	0.03	0.01	0.05	0.04	0.03
**810**	0.05	0.00	0.01	0.01	0.01	0.08	0.04	0.01
**814**	0.02	0.01	0.03	0.03	0.01	0.06	0.05	0.02
**817**	0.01	0.02	0.04	0.04	0.02	0.06	0.05	0.03
**820**	0.00	0.01	0.04	0.04	0.03	0.08	0.02	0.00
**823**	0.02	0.03	0.06	0.07	0.02	0.05	0.01	0.01
**826**	0.01	0.04	0.06	0.07	0.00	0.05	0.02	0.02
**830**	0.01	0.04	0.07	0.08	0.00	0.05	0.05	0.04
**833**	0.03	0.05	0.07	0.08	0.03	0.04	0.04	0.04
**836**	0.03	0.05	0.07	0.08	0.04	0.03	0.06	0.05
**839**	0.01	0.05	0.07	0.08	0.05	0.03	0.07	0.07
**843**	0.01	0.05	0.05	0.06	0.07	0.05	0.07	0.06
**846**	0.01	0.05	0.05	0.06	0.07	0.04	0.08	0.08
**849**	0.02	0.05	0.05	0.06	0.08	0.05	0.07	0.07
**852**	0.01	0.05	0.04	0.05	0.09	0.05	0.08	0.08
**856**	0.02	0.05	0.04	0.05	0.09	0.05	0.08	0.08
**859**	0.01	0.05	0.04	0.05	0.09	0.06	0.08	0.07
**862**	0.01	0.05	0.04	0.05	0.10	0.06	0.08	0.08
**865**	0.02	0.04	0.03	0.04	0.10	0.07	0.07	0.06
**868**	0.07	0.07	0.05	0.06	0.12	0.03	0.06	0.07
**872**	0.04	0.07	0.06	0.07	0.12	0.03	0.08	0.08
**875**	0.02	0.06	0.06	0.06	0.11	0.05	0.08	0.08
**878**	0.02	0.06	0.04	0.05	0.11	0.06	0.07	0.07
**881**	0.08	0.07	0.04	0.04	0.14	0.04	0.07	0.07
**885**	0.04	0.05	0.02	0.03	0.12	0.06	0.08	0.07
**888**	0.05	0.04	0.00	0.00	0.13	0.07	0.08	0.06
**891**	0.07	0.06	0.03	0.04	0.10	0.04	0.09	0.09
**894**	0.07	0.03	0.01	0.01	0.12	0.07	0.08	0.07
**897**	0.12	0.03	0.01	0.01	0.09	0.06	0.08	0.05
**901**	0.08	0.03	0.02	0.01	0.07	0.06	0.06	0.04
**904**	0.10	0.04	0.04	0.04	0.04	0.05	0.05	0.04
**907**	0.09	0.07	0.08	0.08	0.02	0.02	0.02	0.03
**910**	0.13	0.05	0.04	0.04	0.05	0.04	0.00	0.01
**913**	0.09	0.04	0.04	0.04	0.01	0.04	0.02	0.02
**917**	0.15	0.03	0.00	0.00	0.03	0.08	0.01	0.02
**920**	0.16	0.04	0.01	0.01	0.04	0.05	0.01	0.01
**923**	0.12	0.04	0.04	0.04	0.01	0.05	0.03	0.02
**926**	0.10	0.02	0.04	0.03	0.01	0.04	0.07	0.05
**930**	0.18	0.09	0.07	0.07	0.04	0.09	0.00	0.02
**933**	0.05	0.02	0.02	0.02	0.09	0.04	0.08	0.04
**936**	0.10	0.06	0.12	0.09	0.21	0.06	0.14	0.17

**939**	0.01	0.02	0.17	0.15	0.25	0.09	0.06	0.01
**942**	0.16	0.06	0.20	0.21	0.27	0.16	0.05	0.06
**946**	0.15	0.04	0.18	0.17	0.20	0.17	0.07	0.06
**949**	0.23	0.03	0.17	0.17	0.24	0.08	0.12	0.12
**952**	0.08	0.07	0.01	0.03	0.13	0.01	0.09	0.03
**955**	0.09	0.02	0.05	0.05	0.20	0.15	0.13	0.12
**958**	0.10	0.05	0.08	0.09	0.07	0.13	0.05	0.02
**961**	0.20	0.07	0.08	0.09	0.16	0.02	0.02	0.00
**965**	0.05	0.08	0.19	0.20	0.05	0.02	0.00	0.03
**968**	0.05	0.02	0.17	0.20	0.03	0.10	0.01	0.01
**971**	0.04	0.03	0.11	0.12	0.06	0.17	0.01	0.03
**974**	0.07	0.02	0.22	0.23	0.08	0.09	0.11	0.08
**977**	0.14	0.05	0.11	0.13	0.02	0.20	0.04	0.02
**981**	0.06	0.02	0.19	0.20	0.10	0.16	0.12	0.08
**984**	0.03	0.04	0.21	0.21	0.15	0.19	0.13	0.09
**987**	0.00	0.03	0.19	0.21	0.13	0.21	0.09	0.06
**990**	0.12	0.01	0.28	0.29	0.12	0.12	0.08	0.09
**993**	0.12	0.06	0.19	0.19	0.16	0.24	0.07	0.02
**996**	0.28	0.05	0.20	0.19	0.10	0.19	0.03	0.03
**1000**	0.33	0.09	0.15	0.14	0.02	0.31	0.02	0.06

Supplementary Table 8 Important wavelengths according the PLSR analysis at dough ripeness. Red = high influence, Green = low influence

**Wavelength**	DW ears	DW leaves	DW leaf sheaths	DW culm	DW total	N uptake ears	N uptake leaves	N uptake leaf sheaths	N uptake culm	N uptake total
**401**	0.03	0.02	0.04	0.00	0.01	0.04	0.12	0.06	0.07	0.00
**404**	0.03	0.02	0.04	0.00	0.01	0.04	0.12	0.05	0.07	0.00
**408**	0.03	0.02	0.05	0.00	0.00	0.05	0.12	0.05	0.07	0.00
**411**	0.03	0.02	0.05	0.01	0.00	0.05	0.12	0.04	0.06	0.01
**414**	0.03	0.01	0.05	0.01	0.00	0.05	0.12	0.04	0.06	0.01
**418**	0.03	0.02	0.05	0.01	0.00	0.05	0.12	0.04	0.06	0.02
**421**	0.03	0.02	0.06	0.01	0.00	0.05	0.12	0.03	0.06	0.01
**424**	0.03	0.02	0.05	0.01	0.00	0.05	0.13	0.03	0.06	0.01
**427**	0.02	0.02	0.05	0.01	0.01	0.05	0.13	0.03	0.06	0.01
**431**	0.02	0.03	0.05	0.01	0.01	0.05	0.13	0.03	0.06	0.00
**434**	0.02	0.03	0.05	0.00	0.01	0.04	0.13	0.03	0.07	0.00
**437**	0.02	0.03	0.05	0.00	0.01	0.04	0.13	0.03	0.07	0.01
**441**	0.01	0.03	0.05	0.00	0.01	0.04	0.13	0.03	0.07	0.02
**444**	0.01	0.04	0.04	0.01	0.02	0.03	0.14	0.04	0.07	0.03
**447**	0.01	0.04	0.04	0.01	0.02	0.03	0.14	0.04	0.07	0.03
**450**	0.01	0.04	0.04	0.01	0.02	0.03	0.14	0.04	0.07	0.03
**454**	0.00	0.04	0.04	0.01	0.02	0.02	0.14	0.04	0.07	0.04
**457**	0.00	0.04	0.04	0.01	0.03	0.02	0.14	0.04	0.07	0.05
**460**	0.01	0.04	0.04	0.01	0.03	0.02	0.14	0.04	0.07	0.05
**464**	0.01	0.04	0.04	0.02	0.03	0.02	0.13	0.04	0.07	0.05
**467**	0.01	0.04	0.03	0.02	0.03	0.01	0.13	0.04	0.07	0.05
**470**	0.01	0.04	0.03	0.01	0.03	0.01	0.13	0.04	0.06	0.05
**473**	0.01	0.04	0.03	0.02	0.03	0.01	0.13	0.04	0.06	0.05
**477**	0.02	0.04	0.03	0.02	0.03	0.01	0.13	0.04	0.06	0.05
**480**	0.02	0.04	0.03	0.02	0.03	0.01	0.13	0.05	0.06	0.06
**483**	0.02	0.04	0.02	0.02	0.03	0.00	0.13	0.05	0.06	0.06
**486**	0.02	0.04	0.02	0.03	0.03	0.00	0.13	0.05	0.07	0.07
**490**	0.03	0.04	0.02	0.03	0.03	0.00	0.13	0.05	0.07	0.07
**493**	0.03	0.04	0.01	0.03	0.04	0.01	0.13	0.05	0.07	0.08
**496**	0.03	0.04	0.01	0.03	0.04	0.01	0.12	0.05	0.07	0.08
**500**	0.03	0.03	0.01	0.03	0.04	0.01	0.12	0.05	0.07	0.09
**503**	0.03	0.03	0.01	0.03	0.04	0.01	0.11	0.05	0.06	0.09
**506**	0.03	0.02	0.02	0.02	0.04	0.00	0.11	0.05	0.05	0.09
**509**	0.03	0.01	0.03	0.01	0.04	0.00	0.10	0.05	0.04	0.08
**513**	0.02	0.01	0.03	0.01	0.03	0.01	0.09	0.05	0.03	0.08
**516**	0.02	0.02	0.05	0.02	0.03	0.02	0.08	0.05	0.01	0.08

**519**	0.02	0.04	0.06	0.04	0.03	0.02	0.07	0.05	0.01	0.08
**523**	0.00	0.07	0.07	0.07	0.03	0.04	0.05	0.05	0.01	0.05
**526**	0.00	0.09	0.09	0.08	0.05	0.03	0.05	0.05	0.02	0.01
**529**	0.01	0.11	0.10	0.10	0.08	0.02	0.05	0.05	0.05	0.03
**532**	0.03	0.13	0.11	0.12	0.10	0.00	0.05	0.05	0.07	0.06
**536**	0.03	0.14	0.12	0.14	0.11	0.01	0.05	0.05	0.09	0.08
**539**	0.03	0.16	0.13	0.16	0.12	0.01	0.03	0.05	0.08	0.10
**542**	0.04	0.17	0.14	0.17	0.13	0.02	0.03	0.04	0.09	0.11
**546**	0.04	0.18	0.14	0.18	0.13	0.03	0.02	0.04	0.10	0.12
**549**	0.05	0.19	0.14	0.19	0.14	0.04	0.01	0.04	0.11	0.13
**552**	0.05	0.19	0.14	0.19	0.15	0.05	0.01	0.04	0.12	0.12
**555**	0.04	0.19	0.13	0.18	0.14	0.04	0.01	0.04	0.11	0.10
**559**	0.04	0.18	0.13	0.17	0.14	0.03	0.02	0.04	0.11	0.08
**562**	0.02	0.17	0.12	0.16	0.12	0.01	0.02	0.04	0.11	0.06
**565**	0.00	0.16	0.12	0.14	0.10	0.01	0.02	0.03	0.10	0.03
**569**	0.02	0.14	0.12	0.12	0.07	0.04	0.02	0.02	0.08	0.01
**572**	0.05	0.13	0.11	0.10	0.04	0.06	0.01	0.02	0.05	0.05
**575**	0.06	0.11	0.10	0.09	0.00	0.08	0.01	0.01	0.02	0.09
**578**	0.06	0.09	0.09	0.08	0.02	0.09	0.00	0.01	0.01	0.12
**582**	0.05	0.07	0.08	0.07	0.03	0.08	0.00	0.01	0.04	0.15
**585**	0.05	0.06	0.08	0.06	0.05	0.08	0.00	0.00	0.04	0.15
**588**	0.05	0.05	0.07	0.05	0.05	0.08	0.00	0.00	0.04	0.15
**592**	0.05	0.05	0.07	0.05	0.05	0.08	0.00	0.00	0.03	0.13
**595**	0.05	0.05	0.06	0.04	0.06	0.08	0.00	0.00	0.03	0.13
**598**	0.05	0.04	0.06	0.04	0.06	0.07	0.01	0.01	0.03	0.12
**601**	0.05	0.04	0.05	0.04	0.06	0.07	0.01	0.01	0.03	0.11
**605**	0.05	0.03	0.05	0.03	0.06	0.07	0.01	0.01	0.03	0.10
**608**	0.04	0.03	0.04	0.02	0.06	0.06	0.00	0.02	0.02	0.10
**611**	0.04	0.02	0.03	0.02	0.07	0.05	0.01	0.02	0.02	0.09
**615**	0.03	0.01	0.02	0.01	0.07	0.05	0.00	0.02	0.01	0.08
**618**	0.04	0.00	0.02	0.00	0.07	0.05	0.00	0.02	0.01	0.07
**621**	0.03	0.00	0.01	0.01	0.07	0.04	0.00	0.02	0.00	0.06
**624**	0.03	0.01	0.00	0.02	0.08	0.03	0.00	0.02	0.00	0.05
**628**	0.02	0.01	0.00	0.02	0.08	0.03	0.01	0.03	0.01	0.04
**631**	0.02	0.02	0.00	0.02	0.08	0.02	0.01	0.03	0.00	0.03
**634**	0.02	0.03	0.00	0.03	0.08	0.02	0.01	0.03	0.01	0.02
**637**	0.01	0.03	0.01	0.03	0.08	0.01	0.01	0.03	0.01	0.00
**641**	0.01	0.04	0.01	0.04	0.09	0.00	0.01	0.03	0.02	0.00
**644**	0.00	0.05	0.02	0.05	0.09	0.01	0.00	0.02	0.03	0.01
**647**	0.01	0.06	0.04	0.07	0.09	0.02	0.00	0.02	0.04	0.01
**651**	0.02	0.08	0.05	0.08	0.08	0.04	0.01	0.01	0.06	0.01
**654**	0.04	0.09	0.06	0.09	0.08	0.05	0.02	0.00	0.07	0.01
**657**	0.05	0.10	0.08	0.11	0.07	0.07	0.03	0.01	0.08	0.02
**660**	0.07	0.12	0.10	0.13	0.05	0.09	0.04	0.02	0.09	0.02

**664**	0.09	0.13	0.12	0.14	0.04	0.11	0.04	0.04	0.10	0.02
**667**	0.11	0.15	0.14	0.16	0.03	0.13	0.05	0.05	0.11	0.03
**670**	0.12	0.16	0.16	0.17	0.02	0.15	0.06	0.07	0.12	0.02
**673**	0.13	0.17	0.17	0.18	0.01	0.16	0.06	0.07	0.12	0.04
**677**	0.14	0.17	0.18	0.19	0.01	0.17	0.05	0.08	0.12	0.04
**680**	0.14	0.17	0.18	0.18	0.01	0.17	0.05	0.08	0.11	0.06
**683**	0.13	0.16	0.17	0.17	0.02	0.16	0.04	0.07	0.09	0.08
**687**	0.11	0.14	0.15	0.15	0.02	0.14	0.02	0.05	0.06	0.09
**690**	0.08	0.11	0.12	0.11	0.04	0.10	0.02	0.02	0.01	0.10
**693**	0.04	0.08	0.08	0.07	0.04	0.06	0.06	0.01	0.05	0.10
**696**	0.00	0.05	0.04	0.03	0.04	0.02	0.09	0.05	0.10	0.09
**700**	0.04	0.01	0.01	0.01	0.03	0.03	0.13	0.07	0.16	0.07
**703**	0.07	0.00	0.01	0.03	0.02	0.06	0.15	0.10	0.21	0.05
**706**	0.11	0.01	0.03	0.04	0.00	0.10	0.16	0.12	0.24	0.02
**709**	0.14	0.01	0.04	0.04	0.02	0.13	0.16	0.14	0.26	0.00
**713**	0.15	0.01	0.03	0.02	0.06	0.13	0.15	0.15	0.26	0.04
**716**	0.17	0.02	0.02	0.01	0.08	0.14	0.14	0.15	0.27	0.07
**719**	0.20	0.05	0.01	0.04	0.09	0.16	0.12	0.18	0.24	0.07
**722**	0.21	0.08	0.00	0.08	0.10	0.17	0.09	0.20	0.21	0.08
**726**	0.20	0.10	0.01	0.10	0.11	0.16	0.07	0.21	0.17	0.09
**729**	0.18	0.12	0.01	0.13	0.11	0.14	0.03	0.21	0.12	0.09
**732**	0.16	0.12	0.00	0.13	0.10	0.12	0.02	0.20	0.10	0.06
**736**	0.12	0.12	0.01	0.12	0.09	0.08	0.01	0.17	0.07	0.06
**739**	0.08	0.10	0.02	0.11	0.06	0.06	0.01	0.14	0.05	0.04
**742**	0.06	0.08	0.04	0.09	0.02	0.05	0.01	0.12	0.03	0.00
**745**	0.02	0.07	0.04	0.07	0.01	0.01	0.01	0.07	0.04	0.00
**749**	0.02	0.05	0.03	0.05	0.01	0.01	0.00	0.04	0.04	0.00
**752**	0.01	0.03	0.06	0.03	0.07	0.00	0.00	0.04	0.03	0.07
**755**	0.05	0.02	0.03	0.01	0.05	0.04	0.01	0.01	0.06	0.05
**758**	0.03	0.02	0.02	0.02	0.10	0.02	0.02	0.02	0.05	0.07
**762**	0.03	0.01	0.04	0.03	0.11	0.03	0.05	0.01	0.07	0.00
**765**	0.01	0.01	0.02	0.01	0.13	0.01	0.05	0.03	0.05	0.03
**768**	0.03	0.02	0.01	0.01	0.13	0.01	0.03	0.01	0.05	0.06
**771**	0.04	0.01	0.04	0.01	0.11	0.02	0.01	0.01	0.03	0.04
**775**	0.04	0.01	0.04	0.01	0.09	0.03	0.00	0.02	0.03	0.02
**778**	0.04	0.00	0.04	0.00	0.08	0.03	0.01	0.03	0.01	0.01
**781**	0.01	0.01	0.06	0.01	0.09	0.00	0.03	0.01	0.01	0.04
**784**	0.02	0.03	0.05	0.02	0.08	0.01	0.04	0.02	0.01	0.02
**788**	0.02	0.03	0.03	0.03	0.07	0.01	0.04	0.02	0.01	0.00
**791**	0.01	0.02	0.02	0.02	0.08	0.00	0.02	0.01	0.00	0.01
**794**	0.00	0.03	0.02	0.03	0.07	0.00	0.04	0.00	0.02	0.01
**797**	0.00	0.04	0.01	0.04	0.06	0.00	0.05	0.01	0.03	0.03
**801**	0.01	0.04	0.00	0.04	0.04	0.02	0.04	0.02	0.01	0.04

**804**	0.01	0.05	0.00	0.04	0.03	0.01	0.05	0.01	0.02	0.03
**807**	0.02	0.04	0.01	0.04	0.05	0.02	0.04	0.00	0.01	0.01
**810**	0.02	0.04	0.01	0.05	0.04	0.02	0.03	0.01	0.01	0.02
**814**	0.03	0.04	0.04	0.06	0.04	0.01	0.01	0.03	0.01	0.02
**817**	0.03	0.04	0.06	0.07	0.04	0.01	0.02	0.03	0.01	0.08
**820**	0.05	0.04	0.04	0.07	0.06	0.03	0.03	0.06	0.03	0.04
**823**	0.04	0.03	0.03	0.05	0.06	0.03	0.02	0.07	0.03	0.03
**826**	0.01	0.04	0.05	0.06	0.00	0.00	0.03	0.03	0.01	0.10
**830**	0.04	0.04	0.02	0.05	0.02	0.04	0.04	0.05	0.03	0.05
**833**	0.01	0.04	0.04	0.05	0.02	0.01	0.03	0.01	0.00	0.09
**836**	0.04	0.04	0.01	0.04	0.00	0.04	0.04	0.04	0.03	0.04
**839**	0.02	0.04	0.01	0.03	0.03	0.03	0.05	0.02	0.01	0.04
**843**	0.03	0.05	0.04	0.05	0.04	0.05	0.07	0.02	0.03	0.04
**846**	0.02	0.05	0.05	0.04	0.06	0.04	0.08	0.02	0.04	0.06
**849**	0.00	0.06	0.04	0.04	0.08	0.02	0.08	0.01	0.04	0.08
**852**	0.00	0.05	0.05	0.04	0.10	0.02	0.07	0.00	0.02	0.10
**856**	0.01	0.04	0.05	0.03	0.09	0.03	0.07	0.01	0.02	0.09
**859**	0.04	0.04	0.08	0.03	0.07	0.06	0.07	0.04	0.03	0.04
**862**	0.06	0.03	0.10	0.03	0.05	0.08	0.07	0.06	0.04	0.02
**865**	0.04	0.05	0.09	0.03	0.08	0.06	0.09	0.04	0.04	0.04
**868**	0.01	0.04	0.07	0.03	0.11	0.03	0.08	0.02	0.01	0.10
**872**	0.05	0.04	0.10	0.03	0.09	0.08	0.08	0.04	0.04	0.07
**875**	0.06	0.03	0.10	0.02	0.08	0.09	0.08	0.05	0.04	0.04
**878**	0.04	0.04	0.11	0.03	0.09	0.07	0.09	0.04	0.05	0.07
**881**	0.03	0.05	0.09	0.05	0.12	0.06	0.10	0.03	0.04	0.10
**885**	0.02	0.05	0.08	0.04	0.14	0.05	0.07	0.02	0.02	0.09
**888**	0.02	0.07	0.10	0.05	0.15	0.05	0.10	0.02	0.03	0.10
**891**	0.05	0.03	0.09	0.04	0.08	0.08	0.08	0.08	0.03	0.04
**894**	0.07	0.06	0.07	0.07	0.10	0.09	0.06	0.07	0.02	0.03
**897**	0.07	0.04	0.03	0.05	0.08	0.08	0.04	0.10	0.01	0.05
**901**	0.07	0.04	0.01	0.07	0.09	0.08	0.04	0.07	0.00	0.12
**904**	0.06	0.04	0.01	0.06	0.08	0.07	0.02	0.09	0.00	0.11
**907**	0.07	0.01	0.04	0.03	0.07	0.06	0.01	0.10	0.01	0.13
**910**	0.08	0.03	0.02	0.05	0.07	0.09	0.01	0.12	0.01	0.09
**913**	0.06	0.00	0.03	0.04	0.06	0.07	0.00	0.11	0.03	0.13
**917**	0.07	0.03	0.02	0.05	0.07	0.07	0.02	0.10	0.00	0.16
**920**	0.06	0.00	0.03	0.03	0.07	0.07	0.00	0.09	0.04	0.18
**923**	0.01	0.02	0.05	0.01	0.08	0.02	0.01	0.09	0.05	0.19
**926**	0.05	0.02	0.05	0.04	0.08	0.06	0.03	0.14	0.00	0.15
**930**	0.14	0.04	0.07	0.03	0.05	0.14	0.03	0.20	0.02	0.08
**933**	0.14	0.00	0.15	0.09	0.01	0.13	0.02	0.14	0.02	0.20
**936**	0.20	0.13	0.15	0.05	0.27	0.18	0.08	0.36	0.01	0.02
**939**	0.12	0.06	0.21	0.02	0.14	0.10	0.06	0.18	0.04	0.08
**942**	0.12	0.06	0.20	0.05	0.11	0.08	0.09	0.12	0.09	0.01

**946**	0.15	0.04	0.16	0.00	0.16	0.12	0.07	0.14	0.08	0.03
**949**	0.23	0.01	0.14	0.03	0.23	0.20	0.07	0.19	0.03	0.01
**952**	0.06	0.06	0.13	0.08	0.02	0.05	0.00	0.03	0.10	0.00
**955**	0.00	0.00	0.12	0.02	0.10	0.00	0.00	0.10	0.04	0.02
**958**	0.10	0.08	0.02	0.09	0.07	0.12	0.06	0.05	0.05	0.17
**961**	0.07	0.04	0.06	0.06	0.01	0.06	0.04	0.02	0.03	0.07
**965**	0.01	0.07	0.03	0.05	0.04	0.00	0.05	0.01	0.05	0.07
**968**	0.07	0.03	0.03	0.05	0.07	0.09	0.08	0.05	0.05	0.13
**971**	0.03	0.02	0.05	0.02	0.01	0.06	0.07	0.05	0.03	0.07
**974**	0.07	0.05	0.07	0.07	0.02	0.10	0.08	0.01	0.03	0.15
**977**	0.06	0.03	0.03	0.04	0.01	0.05	0.01	0.06	0.03	0.09
**981**	0.03	0.02	0.03	0.01	0.04	0.01	0.03	0.02	0.02	0.09
**984**	0.02	0.01	0.06	0.00	0.05	0.01	0.05	0.03	0.02	0.07
**987**	0.12	0.03	0.09	0.01	0.00	0.07	0.08	0.01	0.01	0.01
**990**	0.15	0.03	0.03	0.05	0.08	0.14	0.03	0.09	0.02	0.08
**993**	0.15	0.09	0.05	0.07	0.01	0.13	0.05	0.04	0.06	0.01
**996**	0.20	0.15	0.01	0.14	0.02	0.20	0.03	0.06	0.02	0.01
**1000**	0.29	0.22	0.06	0.21	0.03	0.29	0.09	0.00	0.03	0.00
